# Phosphoinositide Metabolism Links cGMP-Dependent Protein Kinase G to Essential Ca^2+^ Signals at Key Decision Points in the Life Cycle of Malaria Parasites

**DOI:** 10.1371/journal.pbio.1001806

**Published:** 2014-03-04

**Authors:** Mathieu Brochet, Mark O. Collins, Terry K. Smith, Eloise Thompson, Sarah Sebastian, Katrin Volkmann, Frank Schwach, Lia Chappell, Ana Rita Gomes, Matthew Berriman, Julian C. Rayner, David A. Baker, Jyoti Choudhary, Oliver Billker

**Affiliations:** 1 Wellcome Trust Sanger Institute, Hinxton, Cambridge, United Kingdom; 2 Schools of Biology and Chemistry, Biomedical Sciences Research Complex, The North Haugh, The University of Saint Andrews, St. Andrews, Fife United Kingdom; 3 Faculty of Infectious and Tropical Diseases, London School of Hygiene & Tropical Medicine, London, United Kingdom; Stanford University, United States of America

## Abstract

Many critical events in the *Plasmodium* life cycle rely on the controlled release of Ca^2+^ from intracellular stores to activate stage-specific Ca^2+^-dependent protein kinases. Using the motility of *Plasmodium berghei* ookinetes as a signalling paradigm, we show that the cyclic guanosine monophosphate (cGMP)-dependent protein kinase, PKG, maintains the elevated level of cytosolic Ca^2+^ required for gliding motility. We find that the same PKG-dependent pathway operates upstream of the Ca^2+^ signals that mediate activation of *P. berghei* gametocytes in the mosquito and egress of *Plasmodium falciparum* merozoites from infected human erythrocytes. Perturbations of PKG signalling in gliding ookinetes have a marked impact on the phosphoproteome, with a significant enrichment of *in vivo* regulated sites in multiple pathways including vesicular trafficking and phosphoinositide metabolism. A global analysis of cellular phospholipids demonstrates that in gliding ookinetes PKG controls phosphoinositide biosynthesis, possibly through the subcellular localisation or activity of lipid kinases. Similarly, phosphoinositide metabolism links PKG to egress of *P. falciparum* merozoites, where inhibition of PKG blocks hydrolysis of phosphatidylinostitol (4,5)-bisphosphate. In the face of an increasing complexity of signalling through multiple Ca^2+^ effectors, PKG emerges as a unifying factor to control multiple cellular Ca^2+^ signals essential for malaria parasite development and transmission.

## Abstract 2

Chemical genetics and a global comparative analysis of phosphorylation and phospholipids in vivo shows that PKG is the upstream regulator that induces calcium signals that enables *Plasmodium* to progress through its complex life cycle.

## Author Summary

Malaria, caused by *Plasmodium* spp. parasites, is a profound human health problem. *Plasmodium* parasites progress through a complex life cycle as they move between infected humans and blood-feeding mosquitoes. We know that tight regulation of calcium ion levels within the cytosol of the parasite is critical to control multiple signalling events in their life cycle. However, how these calcium levels are controlled remains a mystery. Here, we show that a single protein kinase, the cGMP-dependent protein kinase G (PKG), controls the calcium signals that are critical at three different points of the life cycle: (1) for the exit of the merozoite form of the parasite from human erythrocytes (red blood cells), (2) for the cellular activation that happens when *Plasmodium* sexual transmission stages are ingested by a blood-feeding mosquito, and (3) for the productive gliding of the ookinete, which is the parasite stage that invades the mosquito midgut. We provide initial evidence that the universal role of PKG relies on the production of lipid precursors which then give rise to inositol (1,4,5)-trisphosphate (IP_3_), a messenger molecule that serves as a signal for the release of calcium from stores within the parasite. This signalling pathway provides a potential target to block both malaria development in the human host and transmission to the mosquito vector.

## Introduction

Malaria is caused by vector-born protozoan parasites of the genus *Plasmodium*, which cycle between mosquitoes and humans. Waves of fever arise from the synchronised egress of merozoites from erythrocytes, an event that must be followed by the invasion of fresh red blood cells (RBCs) for the asexual replicative cycle to continue. Precise timing of egress is crucial for parasite survival as premature or late egress leads to noninvasive merozoites [1]. Parasite transmission to mosquitoes relies on gametocytes, sexual precursor stages that are developmentally arrested in the blood but that resume their development within seconds of being taken up into a mosquito blood meal. Gametocytes respond rapidly to environmental signals including a small mosquito molecule, xanthurenic acid (XA), and a concomitant drop in temperature [2]. Egress of gametes from the host erythrocyte occurs within 10 min of gametocyte ingestion by the mosquito and is followed by fertilisation. Within 24 h zygotes transform into ookinetes, which move actively through the blood meal to colonise the epithelial monolayer of the mosquito midgut. Each successful ookinete transforms into an extracellular cyst that undergoes sporogony. Eventually thousands of sporozoites are released from each cyst and invade the salivary glands of the mosquito. Once transmitted back into another human, they first replicate in the liver before invading the blood stream. This complex life cycle requires a high degree of coordination to allow the parasites to recognise and respond appropriately to stimuli from their environment. However, the underlying signal transduction pathways remain poorly understood.

Reverse genetics and pharmacological studies have identified 3′-5′-cyclic guanosine monophosphate (cGMP) as an important second messenger for regulating the development of malaria parasites. In *Plasmodium*, cGMP levels are tightly controlled at the level of synthesis by two membrane-associated guanylyl cyclases (GCs) and degradation by four cyclic nucleotide phosphodiesterases (PDEs), all of which show stage specificity in their expression [3]. GCα has resisted knockout attempts in the human malaria parasite *Plasmodium falciparum* [4] and in *Plasmodium berghei* [5], a parasite infecting rodents, suggesting GCα is essential in asexual blood stages. In contrast *gcβ* and *pdeδ* could be deleted in asexual blood stages and the mutants revealed critical functions for both enzymes in gametocytes of *P. falciparum* [6] and in ookinetes of *P. berghei*, where the deletion of *gcβ* results in a marked reduction of gliding motility that could be reversed by the additional deletion of *pdeδ* [5], demonstrating a key role for cGMP in regulating ookinete gliding.

The only known downstream target of cGMP in malaria parasites is a cGMP-dependent protein kinase, PKG [7], which according to current evidence is essential in asexual blood stages of *P. falciparum* [6] and *P. berghei* [5]. Work in *Toxoplasma gondii* and *Eimeria tenella*, coccidian parasites that are related to *Plasmodium*, identified PKG as the primary target for two structurally distinct anticoccidal compounds, the trisubstituted pyrrole compound 1 (C1) and the imidazopyridine-based inhibitor compound 2 (C2). Both compounds achieve high selectivity over PKG of humans by exploiting an unusually small gatekeeper residue within the active site of all apicomplexan PKG enzymes [8]. Mutating the threonine gatekeeper residue of apicomplexan PKG to a larger residue renders parasites resistant to both inhibitors. This provided a powerful genetic tool to study PKG function, first in tachyzoites of *Toxoplasma gondii*, where PKG was found to be important for egress from the host cell, secretion of micronemes, and gliding motility [9], and later in *P. falciparum*, where PKG was shown to be important for the initial activation of gametocytes in response to environmental triggers and for replication of asexual blood stages [4],[10]. Inhibition of PKG in *P. falciparum* resulted in the accumulation of mature segmented schizonts, which did not rupture and failed to release merozoites. A C1-insensitive PKG allele also reversed inhibition of schizont rupture, ruling out off-target effects of C1 as responsible. Recently PKG was shown to operate upstream of a Ca^2+^-dependent protein kinase, CDPK5 [11], and to control exocytosis of two secretory organelles, called exonemes and micronemes, which contain proteins essential for merozoite egress [1].

In mammals cGMP regulates diverse and important cellular functions, ranging from smooth muscle contractility [12] to retinal phototransduction [13]. It mediates cellular response to a range of agonists including peptide hormones and nitric oxide [14]. In *Plasmodium* neither the upstream regulators of cGMP signalling have been identified, nor the cellular targets and downstream effector pathways through which PKG regulates the two distinct biological processes that are schizont egress and gametocyte activation. In the present study, we generate *P. berghei* transgenic lines that express a resistant PKG allele. Using a chemical genetic approach we first show that PKG controls the gliding motility that ookinetes rely on to reach and penetrate the midgut epithelium of the mosquito during transmission. We then use a global analysis of protein phosphorylation by quantitative mass spectrometry to identify pathways that operate downstream of PKG in gliding ookinetes. We chose phosphoinositide metabolism as a putative effector pathway for further validation and demonstrate that PKG controls phosphoinositide synthesis including the production of phosphatidylinositol (4,5)-biphosphate (PI(4,5)P_2_), the precursor of inositol (1,4,5)-trisphosphate (IP_3_), whose synthesis triggers mobilisation of intracellular Ca^2+^ [15]. This leads us to hypothesise that a major function for PKG is to control intracellular Ca^2+^ levels in malaria parasites, through the regulation of phosphoinositide metabolism by lipid kinases. This study presents strong evidence in support of this idea by showing in three life cycle stages and two *Plasmodium* species that activation of PKG is critically required to regulate cytosolic Ca^2+^ levels. PKG emerges as a universal regulator that controls ookinete gliding, gametocyte activation, and schizont rupture.

## Results

### PKG Regulates Gliding Motility of Ookinetes

The *pkg* gene appears to be essential in blood stages of *P. berghei* since it could not be disrupted. So far only pharmacological evidence implicates PKG as the effector kinase of cGMP in gliding ookinetes [5]. To facilitate genetic studies in *P. berghei* we replaced *pkg* with a modified allele, *pkg* ^T619Q^-HA, in which the threonine gatekeeper residue was mutated to a larger glutamine residue together with a C-terminal triple HA epitope tag (Figure S1 and Figure S2A). The equivalent gatekeeper mutation in *P. falciparum* PKG confers resistance to the selective inhibitors C1 and C2 [4],[10]. A transgenic control line without the T619Q mutation, *pkg*-HA, was also generated and the resistance marker was removed from both cloned lines by negative selection to enable subsequent genetic modifications (Figure S2A). We observed no effect of the T619Q mutation on asexual growth rate, gametocyte and ookinete formation, midgut oocyst numbers, salivary gland sporozoite numbers, and sporozoite infectivity to mice (Figure S3).

**Figure 1:**
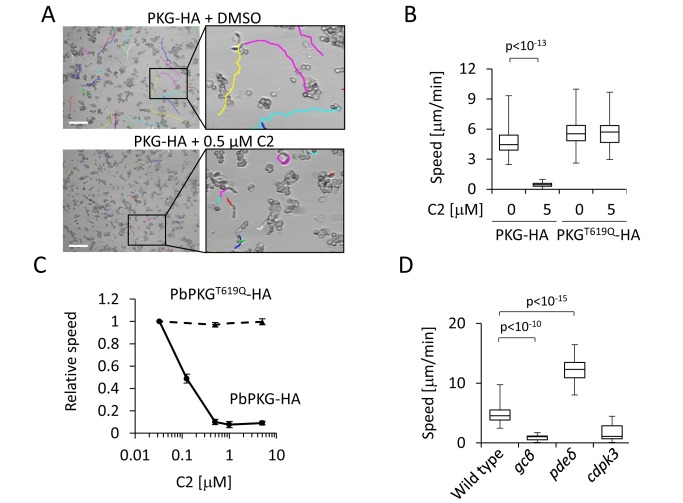
Role of PKG in regulating ookinete gliding. (A) Gliding traces of ookinetes in matrigel recorded for 20 min from a representative field of view. Scale bar, 50 µm. The coloured tracks were created by superimposing individual images from a time series, each marking the tip of each ookinete. (B) Effect of C2 on the gliding speed of ookinetes. (C) Average gliding speed of ookinetes at increasing concentrations of C2. Error bars show standard deviations of 20 ookinetes from each of two independent biological replicates. (D) Gliding speeds of mutant ookinetes. The range of whisker plots in (B) and (D) indicates the 2.5 and 97.5 percentiles, the box includes 50% of all values, and the horizontal line shows median values obtained for 20 ookinetes from each of two independent biological replicates. Statistical analyses in (B) and (D) were carried out using a two-tailed *t* test.

To assess the role of PKG in gliding we recorded time-lapse movies of *in vitro* cultured ookinetes in thin layers of matrigel. Ookinetes expressing PKG-HA were strongly inhibited by C2 (Figure 1A and Figure 1B) with a half-maximal effect of ∼100 nM (Figure 1C). Expression of PKG^T619Q^-HA, in contrast, conferred complete resistance to C2 up to at least 5 µM, demonstrating that PKG is the critical target for C2 and essential for ookinete gliding. Inhibition of PKG by C2 was as potent as disrupting cGMP production genetically by deleting *gcβ* (Figure 1D). In contrast, interfering with degradation of cyclic nucleotides through complete deletion of *pdeδ* (Figure S2B) had the opposite effect, resulting in a marked increase in average gliding speed (Figure 1D). These results show that PKG is a key effector kinase for cGMP in regulating ookinete gliding.

### Identification of Putative PKG-Regulated Pathways by Quantitative Mass Spectrometry

**Figure 2:**
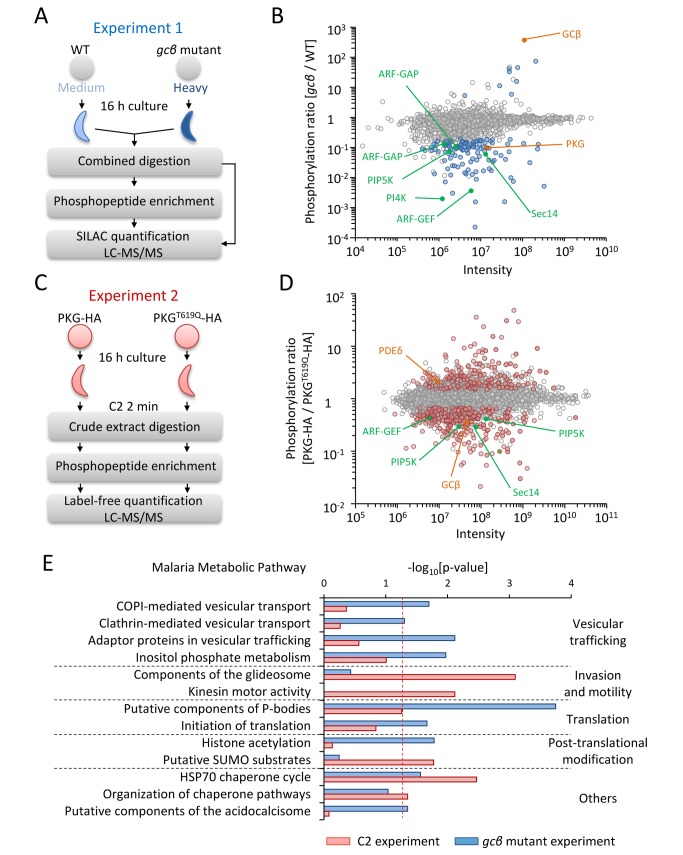
Effects of perturbed cGMP synthesis and PKG inhibition on the ookinete phosphoproteome. (A) Schematic illustrating of experiment 1 to compare global phosphorylation of proteins between wild-type and *gcβ* ookinetes by pulse-chase SILAC labelling with medium (D_4_ L-lysine plus ^13^C_6_ L-arginine) and heavy isotopes (^13^C_6_,^15^N_2_ L-lysine plus ^13^C_6_,^15^N_4_ L-arginine), respectively. Crude extracts from purified ookinetes were combined and analysed together by LC-MS/MS prior or after enrichment for phosphopeptides by IMAC purification. (B) Normalised phosphorylation ratios for all class I sites that were quantified in both wild-type and *gcβ* mutant ookinetes are plotted against the heavy and medium intensities for each site. Data points are coloured to indicate significance of regulation as determined from five biological replicates: blue circles show significantly regulated sites (*p*<0.01, ratio count ≥6, and fold change >3). Labelled sites are in enzymes linked to cGMP signalling (orange) or phosphoinositide metabolism (green). (C) Schematic illustrating experiment 2 to measure the effect of C2 on global protein phosphorylation using label-free quantification. Purified ookinetes expressing PKG-HA or PKG^T619Q^-HA were snap-frozen after a 2 min exposure to C2. (D) Normalised phosphorylation ratios for all class I phosphorylation sites that were quantified in both lines in experiment 2 are plotted against the intensity for each site. Data points are coloured to indicate significance of regulation as determined across six biological replicates: red circles show significantly regulated sites (false discovery rate ≤0.05 and fold change ≥1.5). Proteins with likely roles in cGMP signalling and phosphoinositide metabolism are coloured as in (B). (E) Functional categories from the Malaria Parasite Metabolic Pathway database that were enriched among proteins with regulated phosphorylation sites in experiments 1 (blue bars) or 2 (red bars). The dashed line shows the chosen significance cutoff of *p*<0.05.

In some animal cells, activated PKG can translocate to the nucleus and control transcription [16],[17]. We therefore sequenced mRNA from wild-type and *gcβ* mutant ookinetes but failed to reveal a notable pattern of differential expression (Figure S4A and Figure S4B), suggesting PKG does not regulate gene expression in ookinetes. We next designed two experiments to measure the effect of altered cGMP signalling on the global phosphorylation state of ookinete proteins using mass spectrometry. In the first experiment, we looked for long-term molecular changes in nonmotile *gcβ* mutant parasites as compared to gliding wild-type ookinetes (Figure 2A). We used triplex stable isotope labelling in culture (SILAC) to measure differences between wild-type (medium label) and mutant parasites (heavy label) from five biological replicates. By performing SILAC-based quantitative proteome profiling on 1% of the material, we first identified labelled tryptic peptides from 1,312 proteins. Of these proteins, 763 could be quantified with high stringency, which revealed no notable differences between the mutant and the wild-type proteomes (Figure S4C and Table S1), suggesting the overall protein composition of the *gcβ* mutant was normal. To compare phosphorylation patterns we next performed SILAC-based quantitative phosphoproteomics using the remaining material from each replicate (Figure 2A). Analysing phosphopeptides enriched by immobilised metal ion chromatography (IMAC), we identified 6,375 phosphorylation sites, 5,002 of which were detected with high confidence (class I sites according to [18]). Only 96 class I sites exhibited significantly altered phosphorylation in the *gcβ* mutant as compared with wild-type ookinetes (Figure 2B). Table S1 lists ookinete proteins and their phosphorylation sites, and Table S2 shows the significantly regulated sites in the *gcβ* mutant.

In a second experiment, we asked which ookinete proteins show rapid changes in phosphorylation when PKG is inhibited by C2 for 2 min (Figure 2C). For this experiment, we exposed ookinetes expressing either PKG-HA or PKG^T619Q^-HA to 0.5 µM C2, which blocks gliding only in PKG-HA parasites. Recognising that in the first experiment SILAC had potentially favoured the identification of regulated sites in the part of the proteome that turns over most rapidly and thus incorporates more isotope label [19], we now opted for a label-free strategy. We detected an even larger number of 1,634 phosphorylated proteins, on which we mapped 7,277 unique phosphorylation sites with high confidence (Table S1). Data from six biological replicates lead us to conclude that 266 sites belonging to 193 different proteins were reproducibly regulated in response to inhibition of PKG (Figure 2D and Table S2).

A significantly less phosphorylated site (6-fold, *p* = 0.03) in *gcβ* ookinetes was serine S694 in the activation loop of the kinase catalytic domain of PKG itself. Activation loop phosphorylation is a common mechanism for regulating protein kinase activity, including in mammalian PKG [20]. Down-regulation of S694 probably reflects a state of reduced PKG activity, as was expected in the *gcβ* mutant. In contrast, phosphorylation of PKG S694 was not affected within 2 min of adding C2, suggesting the kinetics of PKG dephosphorylation is slow. However, C2 reduced phosphorylation of S2072 in GCβ and increased phosphorylation of S310 in PDEδ, suggesting possible mechanisms for rapid feedback regulation of cGMP levels, as happens in mammalian cells [21],[22], reinforcing the notion that these enzymes act in the same pathway as PKG to regulate ookinete gliding. Inhibition of PKG also resulted in a rapid 6-fold reduction in the phosphorylation of S11 in the N-terminal leader peptide upstream of the kinase domain of CDPK3, a Ca^2+^-dependent protein kinase important for ookinete gliding [23], indicating its function is linked closely with PKG.

To identify mechanisms of regulation by PKG we asked which cellular pathways were enriched among the proteins with regulated phosphorylation sites (Figure 2E, see Table S2 for gene IDs and site information). Treatment with C2 had the greatest impact on phosphorylation sites of inner membrane complex (IMC) proteins and components of the gliding motor, such as the two glideosome-associated proteins GAP45 and GAPM2, and IMC1b. Microtubule-associated proteins were also enriched, including several dynein and kinesin-related putative motor proteins of unknown function. In marked contrast, regulated phosphosites in the *gcβ* mutant were most abundant in mRNA-interacting proteins involved in splicing and 3′ polyadenylation, and in components of *Plasmodium* P-bodies, such as the RNA helicase, DOZI (development of zygote inhibited), and a putative trailer hitch homolog, CITH, which are both essential for ookinete formation by stabilising translationally repressed mRNAs in the female gametocyte [24]. Also deregulated were multiple sites in a family of Alba domain-containing proteins that form part of the DOZI snRNP complex [24]. Furthermore, regulated phosphoproteins in the *gcβ* mutant were enriched for components of clathrin and COPI-coated vesicles, including a putative clathrin coat assembly protein, AP180, the beta subunit of the coatomer complex, as well as a number of putative regulators of vesicular trafficking, which together point to an important role for protein trafficking in ookinete gliding. Finally, the *gcβ* mutant had a notable abundance of regulated phosphorylation sites in enzymes involved in the metabolism of inositol phospholipids and their regulators. Importantly, many representatives from this group of proteins were also regulated in response to C2 (highlighted in Figure 2B and Figure 2D), suggesting they may be more direct targets of PKG than some of the other regulated phosphoproteins.

### PKG Controls PI4P and PI(4,5)P_2_ Levels in Motile *P. berghei* Ookinetes

**Figure 3:**
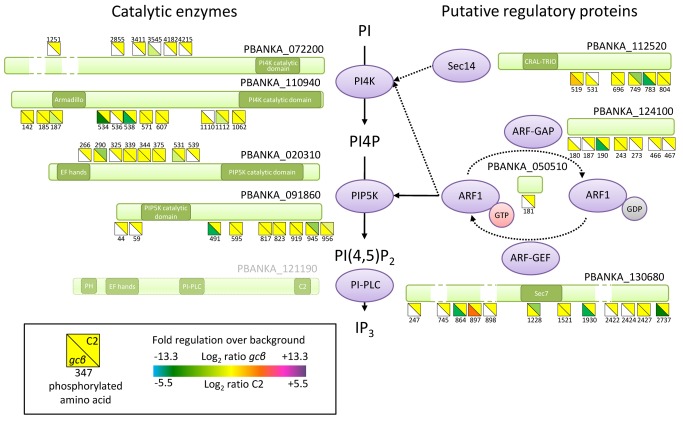
Phosphorylation sites in proteins with likely roles in phosphoinositide metabolism. All class I phosphorylation sites are shown as squares next to the schematic illustrations of the relevant proteins and their annotated functional domains. PI-PLC was not detected and is shown in light green. Each phosphorylation site is represented by a divided square, the colour of which shows the degree of regulation upon inhibition of PKG by C2 or in the *gcβ* mutant. Failure to quantify a phosphorylation site with one of the two experimental designs is shown in white.

Given that enzymes in the inositol phospholipid biosynthetic pathway were identified by both phosphoproteomic approaches, we chose to investigate this pathway in more detail. Phosphorylated phosphatidylinositol lipids have important roles in vesicle trafficking and as a source of secondary messengers in signal transduction. Their biosynthesis from phosphatidyl-1D-*myo*-inositol (PI) is mediated by lipid kinases. The *P. berghei* genome encodes four putative lipid kinases to convert PI first to phosphatidylinositol 4-phosphate (PI4P) and then to phosphatidylinositol (4,5)-bisphosphate (PI(4,5)P_2_) (Figure 3). Hydrolysis of the latter by a PI-specific phospholipase C (PI-PLC) gives rise to the secondary messenger inositol (1,4,5)-trisphosphate (IP_3_), which plays an important role in *P. berghei* gametocytes, where it is responsible for the mobilisation of Ca^2+^ from internal stores, leading to activation and gametogenesis [25]. All four PI kinases were detected in the ookinete phosphoproteome and three contained sites that were less phosphorylated upon inhibition of PKG or disruption of *gcβ* (Figure 3). An important regulator of phosphoinositide metabolism and membrane trafficking is the phosphatidylinositol transfer protein Sec14 [26], the phosphorylation of which was reduced in closely adjacent sites in both experiments. PIP5K activity of the *P. falciparum* orthologue of PBANKA_020310 is controlled by a small G protein of the ADP-ribosylation factor (ARF) family [27], which cycles between an inactive GDP-bound and an active GTP-bound form. In other eukaryotes, the active state of ARF results from its interaction with a guanine nucleotide exchange factor (ARF-GEF) that forces ARF to adopt a new GTP molecule in place of a bound GDP, whereas the inactive state results from hydrolysis of GTP facilitated by a GTPase activating protein, ARF-GAP. Putative ARF-GEF and ARF-GAP proteins are encoded in the *P. berghei* genome, and these also have GCβ/PKG-dependent phosphosites (Figure 3). Taken together these data led us to hypothesise that phosphoinositide metabolism is important for ookinete gliding and regulated by PKG.

**Figure 4:**
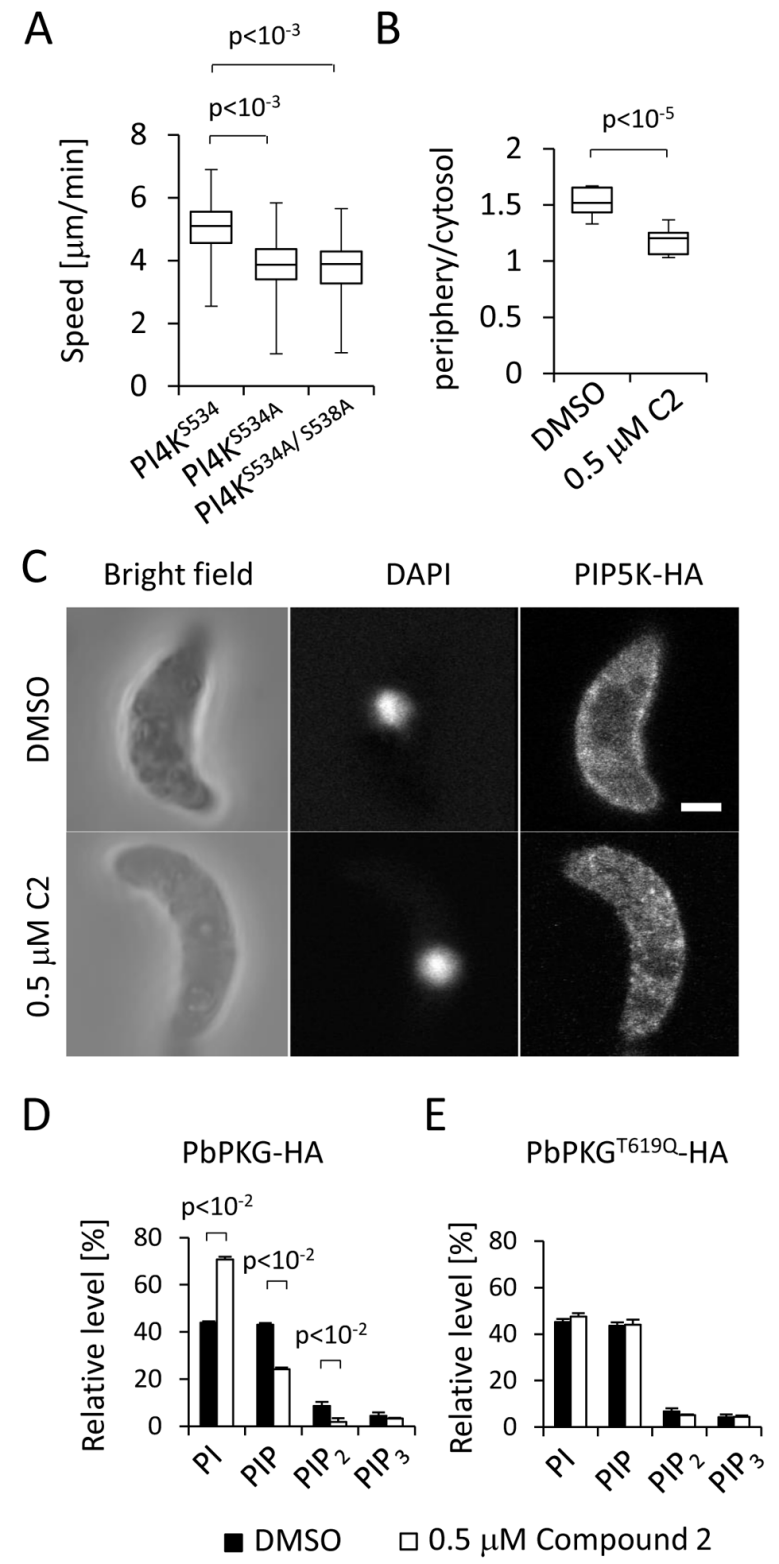
Phosphoinositide phosphorylation links PKG to gliding in *P. berghei* ookinetes. (A) Ookinete gliding speed of PI4K^S534A^ and PI4K^S534A/S538A^ ookinete mutants. Values are representative of 20 individual ookinetes from two independent biological replicates. (B) Ratio of peripheral to cytosolic fluorescence intensity from optical sections taken of different ookinetes from the experiment shown in (C); *n* = 10 sections from different ookinetes. Statistical analysis was carried out using a two-tailed *t* test. (C) Confocal immunofluorescence images of fixed ookinetes showing the effect of 0.5 µM C2 on the cellular distribution of a C-terminally HA tagged PIP5K (PBANKA_020310) expressed from its endogenous promoter. Scale bar, 5 µm. (D) Relative quantification of PI, PIP, PIP_2_, and PIP_3_ levels after 10 min treatment with 0.5 µM C2 or DMSO in PKG-HA ookinetes. (E) As in (D) but for PKG^T619Q^-HA ookinetes. Error bars in (D) and (E) show standard deviations of two biological replicates. The *p* values are from two-tailed *t* test. See also Figure S5.

To test whether the PKG-dependent phosphorylation of enzymes associated with phosphoinositide metabolism has a direct role in ookinete motility, we used experimental genetics to infer the role of putative PI kinases. Both the putative PI4K (PBANKA_110940) and the putative PIP5K (PBANKA_020310) were unable to be genetically disrupted (unpublished data), suggesting these genes may be essential for asexual growth, although both loci could be modified (Figure S2C and Figure S2D). One of the most strongly down-regulated phosphorylation sites in the *gcβ* mutant was S534 of PI4K. To assess the importance of this residue, we generated allelic replacement constructs to mutate S534 to alanine, either on its own or in combination with a nearby phosphorylation site, S538 (Figure S2D). At the ookinete stage, *pi4k^S534A^* and *pi4k^S534A/S538A^* clonal mutants showed a significant decrease in gliding speed compared with a control line, *pi4k^S534^* (Figure 4A and Figure S2D), which would be consistent with phosphorylation of S534 in PI4K contributing to the regulation of phosphoinositide metabolism *in vivo*. A direct link between PKG and phosphoinositide metabolism was also supported by the location of PIP5K-HA, which rapidly redistributed from the cell periphery to the ookinete cytosol in ookinetes treated with 0.5 µM C2 (Figure 4B and Figure 4C).

To define more precisely the role of PKG in regulating phosphoinositide metabolism, we examined the effect of C2 on the phospholipid composition of gliding ookinetes. Analysing total lipid extracts from purified ookinetes by mass spectrometry, we detected PI, phosphatidylcholine (PC), phosphatidylethanolamine (PE), phosphatidylglycerol (PG), cardiolipin (CL), and several other minor phospholipids. For all of these we then determined changes in their relative abundance upon inhibition of PKG (Figure S5A and Figure S5B). Exposing gliding ookinetes to 0.5 µM C2 for 10 min resulted in a marked relative increase in PI, while peaks corresponding to PIP and PIP_2_ molecular species were reduced (Figure 4D). No significant differences in PC, PE, CL, or PG were detected (unpublished data). In control experiments, C2 had no effect on phospholipid composition of ookinetes expressing PKG^T619Q^-HA (Figure 4E). These data suggest a link between PKG and PI4P synthesis and therefore probably with Ca^2+^ signalling in ookinetes. We therefore examined next whether PKG is a positive regulator of Ca^2+^ release.

### PKG Activity Maintains High Cytosolic Ca^2+^ Levels in *P. berghei* Ookinetes

**Figure 5:**
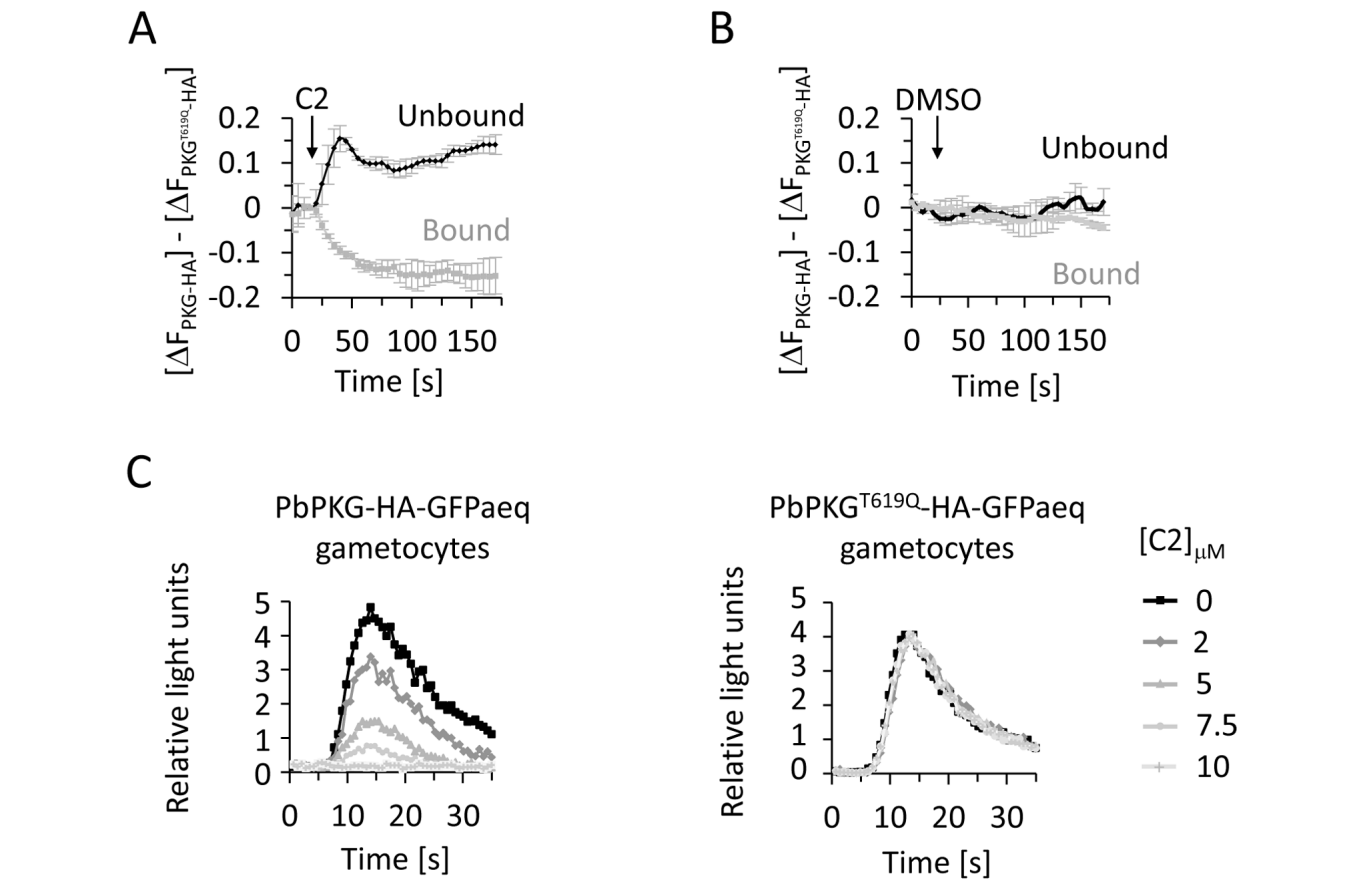
PKG controls cytosolic Ca^2+^ levels in ookinete and upon gametocyte activation in *P. berghei*. (A and B) Determination of relative cytosolic Ca^2+^ levels in purified ookinetes expressing PKG-HA or PKG^T619Q^-HA using pericam, a dual excitation Ca^2+^ reporter. We added 0.5 µM C2 treatment (A) or DMSO treatment (B) at t = 20 s. Fluorescence was normalised as follows: ΔF = (F_n_−F_20_)/F_20_, in which F_n_ is the fluorescence at t = *n* s; F_20_ is the reference time before addition of C2 or DMSO, and was normalised to the baseline provided by the C2-resistant parasites expressing PKG^T619Q^-HA. Error bars show standard errors from three independent replicates each using 10 ookinetes per condition. See Figure S6A and Figure S6B for a validation of the pericam reporter in *P. berghei*. (C) Luminescence responses of gametocytes expressing GFP-aequorin to different concentrations of C2. Gametocytes were stimulated with 50 µM XA at t = 0 s. Data are representative of at least four independent experiments.

To measure Ca^2+^ levels in life ookinetes, we inserted an expression cassette for the free Ca^2+^ reporter pericam into the redundant *p230p* locus of the PKG-HA and PKG^T619Q^-HA lines (Figure S2E). Pericam is a fusion protein comprising calmodulin, GFP, and the M13 peptide corresponding to the calmodulin-binding domain of skeletal muscle myosin light chain kinase [28]. Binding of Ca^2+^ to the EF hands of calmodulin causes the latter to interact with the M13 peptide, which in turn modulates the fluorescence properties of GFP. We expressed a ratiometric form of pericam, in which Ca^2+^ shifts the excitation peak from 415 to 494 nm. Dual excitation imaging detects changes in Ca^2+^ levels through shifts in the ratio of the Ca^2+^ bound to the unbound form of pericam [28]. Ca^2+^-bound and -unbound pericam were uniformly distributed throughout the ookinete cytosol (Figure S6A) and were sensitive to changes in intracellular Ca^2+^ induced by the Ca^2+^ ionophore ionomycin (Figure S6B). Addition of 0.5 µM C2 to PKG-HA-pericam ookinetes resulted in a rapid shift within 15 s from Ca^2+^-bound to -unbound reporter if compared to C2-resistant PKG^T619Q^-HA-pericam parasites (Figure 5A). There was no such response in the solvent control (Figure 5B). These results demonstrate that PKG activity is critical for high cytosolic Ca^2+^ levels to be maintained in gliding ookinetes.

### PKG Controls Agonist-Induced Ca^2+^ Mobilisation Upon Gametocyte Activation

It remains unknown whether ookinetes glide constitutively *in vivo* or whether their behaviour responds to internal or external stimuli. In contrast, gametocytes, the developmentally arrested sexual precursor stages that circulate in the blood stream in a developmentally arrested form, become activated by well-defined environmental triggers within seconds of being taken up by a feeding mosquito. Activation is mediated by a drop in temperature and the concomitant exposure to a chemical stimulus from the mosquito, XA [2]. These well-defined triggers offer an opportunity to ask how PKG and Ca^2+^ interact in response to physiological agonists. At a permissive temperature XA triggers PI(4,5)P_2_ hydrolysis in *P. berghei* gametocytes, presumably by activation of PI-PLC [25], which results in the rapid mobilisation of Ca^2+^ from internal stores. In *P. falciparum*, on the other hand, XA enhances GC activity in membrane preparations [29], and PKG regulates gametocyte activation [10]. If and how signalling through cGMP and Ca^2+^ are linked during gametocyte activation has not been addressed.

To ask if in *P. berghei* gametocytes PKG regulates Ca^2+^ release in response to XA, we introduced into the *dssu* or *cssu* locus of the marker-free PKG-HA and PKG^T619Q^-HA lines an expression cassette for a reporter protein that is based on the Ca^2+^-dependent photoprotein, aequorin (Figure S2F) [30]. XA triggered a transient luminescence response that peaked rapidly after a characteristic lag phase 10 s after stimulation (Figure 5C), as described previously [31]. In parasites expressing PKG-HA this response was dose-dependently blocked by C2 with an IC_50_ of around 3 µM (Figure 5C). C2 acted through inhibition of PKG, because the PKG^T619Q^-HA-GFPaeq line was completely resistant to even higher concentrations of the inhibitor. We conclude that the rapid activation of PKG within seconds of exposing gametocytes to their natural agonist mediates gametocyte activation through the mobilisation of Ca^2+^ and is therefore most likely a key event for the transmission of *Plasmodium* to the mosquito.

### PKG Controls Phosphoinositide and Ca^2+^ Levels in *P. falciparum* Schizonts

**Figure 6:**
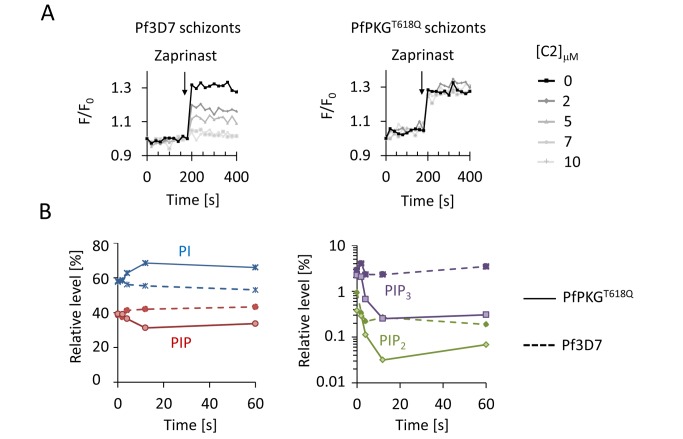
PKG controls phosphoinositide metabolism and Ca^2+^ mobilisation in *P. falciparum* schizonts prior to merozoite egress. (A) Relative fluorescence intensity of ∼10^8^ Fluo-4–loaded synchronised *P. falciparum* schizonts in response to simultaneous exposure to 100 µM zaprinast and increasing concentrations of C2. Data are representative of two independent experiments. (B) Relative abundance over time of PI, PIP (left panel), PIP_2_, and PIP_3_ (right panel, note different scale) after simultaneous inhibition of PDEs by zaprinast and PKG by C2. The response of *P. falciparum* 3D7 (dashed lines) is compared to a transgenic clone expressing the C2-resistant PKG^T618Q^ allele (solid lines).

To explore whether Ca^2+^ release via PKG signalling regulates blood stages in *Plasmodium* parasites, we turned to the major human pathogen *P. falciparum*. In asexual blood stages of *P. falciparum* PKG is critically required for schizonts to rupture and for merozoites to egress [4]. C1 and C2 were shown to block schizont rupture by inhibiting PKG, while conversely, an inhibitor of cGMP-PDE, zaprinast, raises cellular cGMP levels and triggers premature egress through the rapid discharge of micronemes and exonemes from the intracellular parasite that is strictly dependent on parasite PKG [1]. Since discharge of secretory organelles by *Plasmodium* schizonts also depends on Ca^2+^ we asked whether activation of PKG by zaprinast triggers egress by controlling intracellular Ca^2+^ levels. In synchronised *P. falciparum* schizonts loaded with the fluorescent Ca^2+^ sensor Fluo-4, addition of 100 µM zaprinast led to a marked increase in free cytosolic Ca^2+^ within 20 s (Figure S6C). This rapid Ca^2+^ response was mediated by PKG, because it could be inhibited by the simultaneous administration of C2 with a half-maximal effect of ∼2 µM (Figure 6A). Importantly, C2 was completely ineffective in blocking zaprinast-induced Ca^2+^ release in parasites expressing a resistant T618Q allele of PfPKG.

To ask if PKG controlled schizont Ca^2+^ mobilisation by regulating phosphoinositide metabolism, we studied the schizont lipidome (Figure S5C). Zaprinast triggered a rapid depletion of PIP, PIP_2_, and PIP_3_, and a concomitant rise in PI from total lipid extracts within 10 s, consistent with the kinetics of Ca^2+^ mobilisation by the same treatment. Pre-exposing infected erythrocytes to 1 µM C2 blocked the zaprinast-induced depletion of phosphorylated PIs, but only in parasites expressing wild-type PKG and not in parasites expressing the C2-resistant T618Q allele of PKG (Figure 6B). Uninfected erythrocytes, in contrast, contained very little phosphorylated PIs (Figure S5D). Taken together, these data show that activation of PKG by the PDE inhibitor zaprinast triggers a cellular Ca^2+^ response in *P. falciparum* schizonts that is accompanied by the rapid initiation of PIP_2_ hydrolysis.

## Discussion

PKG was discovered in *Eimeria* and *Toxoplasma* as the target for potent anticoccidial inhibitors that achieve selectivity over vertebrate PKG by exploiting the small gatekeeper residue typically found in PKG of apicomplexa, including in malaria parasites [8]. PKG has since been shown to have essential functions in egress, microneme secretion, and gliding of *T. gondii* tachyzoites [9], as well as in biological processes as diverse as merozoite egress and gametocyte activation in *Plasmodium* [1],[10]. As a result PKG is considered as a promising drug target also in malaria parasites [4],[33], yet how PKG performs its wide range of cellular functions has remained elusive.

In this study we demonstrate that PKG is the cGMP effector kinase that regulates gliding of *P. berghei* ookinetes downstream of GCβ and PDEδ. Ookinetes are relatively tractable cells by both biochemical and genetic methods. We therefore used the comparative analysis of total ookinete phosphoproteomes to identify proteins whose phosphorylation is directly or indirectly dependent on PKG. In one type of experiment we compared the abundance of individual phosphopeptides between wild-type and *gcβ* mutant ookinetes. With a second experimental design we assessed the sensitivity of global phosphorylation events in gliding ookinetes to chemical inhibition of PKG by C2. Importantly, by comparing two parasite lines in the presence of C2 that differed in only a single amino acid, the gatekeeper residue of PKG, we could focus our analysis on on-target effects of the inhibitor. Our combined experiments identified >9,000 different phosphorylation sites on nearly 2,000 ookinete proteins. In view of this large number of phosphorylated proteins and the many cellular pathways potentially involved in gliding, it was not surprising to find 250 phosphoproteins reproducibly regulated under either one or both experimental conditions. Statistical pathway enrichment analysis, followed by experimental validation, proved crucial for extracting biological meaning from these complex global data sets. From the significantly enriched cellular pathways downstream of PKG, we selected phosphoinositide metabolism for experimental validation because enzymes in this pathway showed robust signs of differential phosphorylation under both experimental conditions. Importantly, PI(4,5)P_2_ hydrolysis by PI-PLC generates IP_3_, a second messenger that is important for Ca^2+^-dependent gametocyte activation of *P. berghei* [25] and that also elicits Ca^2+^ responses in intraerythrocytic asexual stages of *P. falciparum* [34]. A role for PKG in phosphoinositide metabolism upstream of Ca^2+^ signalling could therefore provide a unifying explanation for the seemingly disparate roles of PKG in different life cycle stages.

### A Universal Role for PKG in Ca^2+^ Mobilisation From Internal Stores Reconciles Its Diverse Functions Across Different *Plasmodium* Species and Stages

All biological processes in apicomplexan parasites known to require PKG are also thought to rely on the release of Ca^2+^ from intracellular stores [11],[31],[32],[35],[36], which in turn activates distinct stage-specific effector pathways including members of a family of plant-like Ca^2+^-dependent protein kinases (CDPKs) [37]. Merozoite egress requires CDPK5 [11], cell cycle progression to S-phase in activated male gametocytes is mediated by CDPK4 [31], and ookinete gliding relies on CDPK3 [23],[35]. By combining a range of genetically encoded and chemical Ca^2+^ reporter systems with PKG gatekeeper mutants in both *P. berghei* and *P. falciparum*, this study demonstrates clearly that PKG controls cytosolic Ca^2+^ levels in all these life cycle stages. The critical importance of PKG upstream of parasite cytosolic Ca^2+^ is thus of universal relevance to parasite development in blood and transmission stages, as it holds true for agonist-induced signalling in gametocytes, in constitutively gliding ookinetes, and after artificial PKG activation triggered by a PDE inhibitor in erythrocytic schizonts.

Cross-talk between second messengers is common in eukaryotic cells. In mammalian vascular smooth muscle cells, for instance, cGMP regulates cytosolic Ca^2+^ negatively, chiefly through PKG1β, which reduces Ca^2+^ release from internal stores by phosphorylating the IP_3_ receptor in the ER membrane [38]. Phototransduction, in contrast, relies on a direct inhibitory interaction of cGMP with a cyclic nucleotide-gated cation channel in the plasma membrane, which appears to have been lost from the apicomplexan genomes during evolution [39]. We here present evidence for a positive interaction between cGMP and Ca^2+^ signalling in malaria parasites that invokes a different mechanism by involving regulation of phosphoinositide metabolism. Studying gametocyte activation in *P. berghei*, we previously identified hydrolysis of PIP_2_ and generation of IP_3_ by PI-PLC as a critical event upstream of Ca^2+^ mobilisation by XA [25]. IP_3_-dependent Ca^2+^ release also operates in *P. falciparum* blood stages [34], although genetic evidence for an IP_3_ receptor in *Plasmodium* is still missing. Because canonical G-protein coupled receptors and heterotrimeric G-proteins, which typically regulate PI-PLC, are absent from the genomes of *Plasmodium* species, it has remained unclear how IP_3_ production in apicomplexan parasites is regulated. Our lipidome analysis of *P. falciparum* schizonts provides strong biochemical evidence that activating PKG with the help of a PDE inhibitor that raises cellular levels of cGMP [1] leads to the rapid and PKG-dependent hydrolysis of PIP_2_ at the same time as cellular Ca^2+^ increases sharply. This would be consistent with a role for PKG for the activation of PI-PLC. Whether this involves phosphorylation remains unknown, as our proteometric studies failed to detect PI-PLC with confidence.

Intriguingly, inhibiting PKG in gliding ookinetes, where Ca^2+^ is already elevated, revealed a different link between PKG and phosphoinositide metabolism. In this situation, inhibiting PKG did not cause PIP_2_ to accumulate, as would be expected if its primary role was to promote PI-PLC activity. Instead, inhibition of PKG revealed phosphorylation of PI as a rate-limiting step. More tentative evidence that in ookinetes PKG regulates signalling at the point of PI phosphorylation comes from two additional observations. First, mutations in phosphorylated serine residues in PI4K reduce ookinete gliding speed. Second, a type I PIP5K of ookinetes localises to the cell periphery in a PKG-dependent manner. Dissociation of PIP5K from the plasma membrane or possibly the IMC of the ookinete could merely be a consequence of substrate depletion [40]. On the other hand, PIP5K, the *P. falciparum* ortholog of which encodes a functional type I PIP5K that is activated by ARF [27], may also provide a more active link to cGMP signalling, as it contains EF-hand-like motifs of a kind typically found in the neuronal Ca^2+^ sensor family of proteins, which intriguingly can function as activators of membrane GCs in other eukaryotes [41].

More work is clearly required to evaluate lipid kinases and PI-PLC as direct substrates of PKG; to establish their precise roles, and those of their products, in linking PKG to Ca^2+^ in *Plasmodium*; and to determine their relative importance in schizonts, ookinetes, and gametocytes. A recent study has proposed that in the mammalian central nervous system PKG regulates presynaptic vesicle endocytosis by controlling PI(4,5)P_2_ synthesis indirectly via the small GTPase RhoA and Rho kinase [42]. However, a different mechanism must operate in apicomplexan parasites, which appear to lack a Rho signalling pathway.

Our identification of PKG as a positive upstream regulator of cytosolic Ca^2+^ levels extends current models of how schizonts rupture in the bloodstream and how XA activates gametocytes in the mosquito. Importantly, it puts a spotlight on the GCs and PDEs that control cGMP levels in the parasite and which must provide the next layer of regulation for some of the key events in the life cycle of malaria parasites.

### Vesicular Trafficking and Microneme Biogenesis

**Figure 7:**
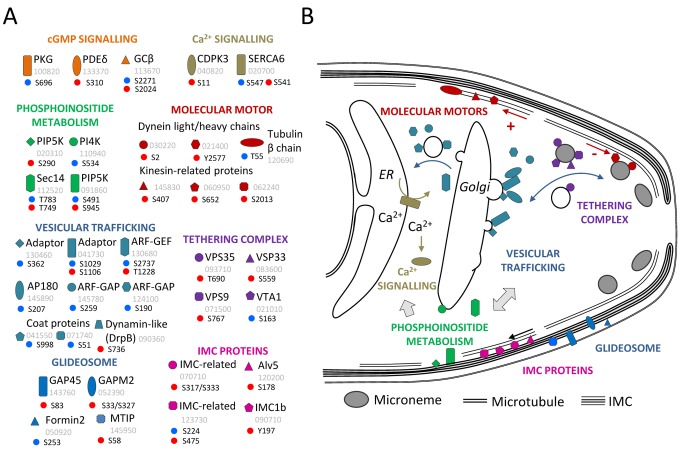
Proteins with PKG-dependent phosphorylation sites and their hypothetical functions in gliding ookinetes. (A) Proteins with phosphorylation sites that are significantly regulated in response to C2 (red dots) or deletion of *gcβ* (blue dots) and that belong either to known signalling pathways are linked to the glideosome, or which belong to the enriched functional groups of proteins with likely roles in vesicular trafficking. The numeric part of the PBANKA gene ID is shown in grey. The amino acid numbers for the regulated sites in each protein are stated next to a letter indicating if the phosphorylated residue is a serine (S), threonine (T), or tyrosine (Y). (B) Model illustrating hypothetical functions for the proteins in (A) in the molecular motor or during microneme biogenesis in a gliding ookinete.

Apicomplexan zoites glide with the help of transmembrane adhesins, which are secreted apically from micronemes, and then translocate posteriorly, where they are shed and left behind in a trail of vesicles that also contain other membrane proteins [43]. Sustained gliding must be fuelled by the continuous synthesis of substantial quantities of proteins and lipids that need to be trafficked to new micronemes, which are then transported to the apical pole of the ookinete for regulated secretion. In the *gcβ* mutant the striking abundance of regulated phosphoproteins with likely functions in vesicular transport (Figure 7A), but also of chaperones (Figure 2E), reflects a profoundly dysregulated secretory system (Figure 7B), which may result from the role of PKG as a regulator of phosphoinositide metabolism in at least two ways. First, the changed phosphorylation pattern in vesicular transport proteins may result from a “traffic jam” following a block in IP_3_/Ca^2+^-dependent regulated secretion of micronemes at the apical end of the ookinete. Second, because in other eukaryotes PIP and PIP_2_ control many essential cellular processes in regulating membrane dynamic and vesicular trafficking [44], PKG-mediated changes in PI phosphorylation could alter vesicular transport more directly and at different stages in the secretory pathway.

Microneme biogenesis is poorly understood in *Plasmodium*, but work in *T. gondii* tachyzoites has identified endosomal sorting signals that traffic micronemal proteins from the Golgi to an endosomal-like compartment before they are packaged into micronemes. In the absence of either dynamin-related protein B (DrpB) or the VPS10/sortilin homolog TgSORTLR, proteins destined for micronemes fail to be targeted from the Golgi to secretory organelles and instead enter the constitutive secretion pathway. TgSORTLR is thought to be an essential cargo receptor to transport microneme and rhoptry proteins to endosomal-like compartments of the *T. gondii* tachyzoite. In other eukaryotes retrograde transport of sortilin to the Golgi relies on its interaction with the conserved retromer complex. We here report regulated phosphorylation for *P. berghei* homologues for another retromer component, VPS35, as well as for DrpB (T690 and S736, respectively; Figure 7A). Similarly, a VPS9 homolog we found regulated in S767 is a likely conserved activator of Rab5 GTPases, which is relevant because in *T. gondii* Rab5A and Rab5C are essential for targeting proteins to unique subsets of micronemes [45]. Although many of the regulated phosphosites we report here are probably not phosphorylated directly by PKG, these examples illustrate that our study provides a rich source of leads for future research into gliding motility and microneme biogenesis in *Plasmodium*.

Our data demonstrate that by regulating phosphoinositide metabolism PKG controls multiple essential cellular processes including critical Ca^2+^ signals and probably vesicular trafficking. Members of a promising class of new antimalarials, the imidazopyrazines, target PI4K [46]. Our analysis provides a rationale for the activity of imidazopyrazines against multiple life cycle stages of *Plasmodium* and highlights how a stage transcending signalling pathway can regulate different critical steps during parasite development. Future work will have to refine the knowledge about the nature of PKG-mediated signalling by identifying direct PKG substrates and the genetic or environmental factors controlling PKG activity.

## Materials and Methods

### Ethics Statement

All animal experiments were conducted under a license from the UK Home Office in accordance with national and European animal welfare guidelines.

### Parasites

*P. berghei* ANKA wild-type strain 2.34 and transgenic lines made in the same background were maintained in female Theiler's Original outbred mice and infections monitored on Giemsa-stained blood films. Exflagellation was quantified 3 d postinfection by adding 4 µl of blood from a superficial tail vein to 150 µl exflagellation medium (RPMI 1640 containing 25 mM HEPES, 4 mM sodium bicarbonate, 5% FCS, 100 µM XA, pH 7.4). Between 13 and 16 min after activation, the number of exflagellating microgametocytes was counted in a haemocytometer and the RBC count determined. The percentage of RBCs containing microgametocytes was assessed on Giemsa-stained smears, and the number of exflagellations per 100 microgametocytes was then calculated. For Ca^2+^ assays, gametocytes were separated from uninfected erythrocytes on a nycodenz cushion made up from 48% of a nycodenz stock (27.6% w/v Nycodenz in 5.0 mM Tris-HCl [pH 7.20], 3.0 mM KCl, 0.3 mM EDTA) and RPMI1640 medium containing 25 mM HEPES, 5% FCS, 4 mM sodium bicarbonate, pH 7.30. Gametocytes were harvested from the interphase.

For ookinete cultures, parasites were maintained in phenyl hydrazine-treated mice. Ookinetes were produced *in vitro* by adding one volume of high gametocyteamia blood in 20 volumes of ookinete medium (RPMI1640 containing 25 mM HEPES, 10% FCS, 100 µM XA, pH 7.5) and incubated at 19°C for 16–18 h. For the *gcβ* mutant experiment, customized RPMI1640 medium (Invitrogen) containing ^13^C_6_,^15^N_2_ L-lysine and ^13^C_6_,^15^N_4_ L-arginine (*gcβ* mutant) or D_4_ L-lysine and ^13^C_6_ L-arginine (wild-type) was used. Conversion efficiency was determined by live staining of ookinetes and activated macrogametes with Cy3-conjugated 13.1 monoclonal antibody against p28. The conversion rate was determined as the number of banana-shaped ookinetes as a percentage of the total number of Cy3-fluorescent cells. For biochemical analysis and Ca^2+^ assays, ookinetes were purified using paramagnetic anti-mouse IgG beads (Life Technologies) coated with anti-p28 mouse monoclonal antibody (13.1). For motility assays, ookinete cultures were added to an equal volume of Matrigel (BDbioscience) containing DMSO or C2 on ice, mixed thoroughly, dropped onto a slide, covered with a cover slip, and sealed with nail polish. After identifying a field containing ookinetes, time-lapse videos were taken (120×; 1 frame every 20 s, for 20 min) on a Leica M205A at 19°C. Movies were analysed with Fiji and the Manual Tracking plugin (http://pacific.mpi-cbg.de/wiki/index.php/Manual_Tracking).

For transmission experiments batches of ∼50 female *Anopheles stephensi*, strain SD500, mosquitoes were allowed to feed on infected mice 3 d after intraperitoneal injection of infected blood. Unfed mosquitoes were removed the day after. Infected mosquitoes were maintained on fructose at 19°C and oocyst numbers were counted on dissected midguts 7 d after feeding. Sporozoite numbers were determined on day 21 by homogenising dissected salivary glands and counting the released sporozoites. To determine sporozoite infectivity to mice 21 d after infection, infected mosquitoes were allowed to feed on naïve mice or 20,000 freshly isolated sporozoites in RPMI 1640 containing 1% penicillin/streptomycin were injected in a volume of 100 µl into the tail vein. Mice were then monitored daily for blood stage parasites.

### Targeting Vector Construction and Transgenic Generation

Tagging, knockout, and allelic replacement constructs were generated using phage recombinase mediated engineering in *Escherichia coli* TSA (Figure S1); *Plasmo*GEM vectors PbG01-2397b11, PbG02_C-11d08, PbG02_C-25e06, and PbG01-2399h12 containing PBANKA_100820 (PKG), PBANKA_110940 (PI4K), PBANKA_020310 (PIP5K), and PBANKA_100820 (PDEä) encoding genes, respectively, (Figure S2) were generated from genomic DNA library clones in linear pJAZZ-OK vectors (Lucigen) as described (Ref. 47 and http://plasmogem.sanger.ac.uk/). Oligonucleotides used are shown in Table S3.

Point mutations were introduced with a two-step strategy using λ Red-ET recombineering in *E. coli*. The first step involved the insertion by homologous recombination of a Zeocin-resistance/Phe-sensitivity cassette surrounded by sequences 5′ and 3′ of the codon of interest amplified using primer pairs specific for the gene of interest (GOI): *goi*-delF/*goi*-delR. Recombinant bacteria were then selected on Zeocin. After verification of the recombination event by PCR, a second round of recombination exchanged the Zeocin-resistance/Phe-sensitivity cassette with a PCR product containing the desired mutation surrounding the codon of interest amplified using *goi*-mutF/*goi*-mutR primer pairs. Bacteria were selected on YEG-Cl kanamycin plates. Mutations were confirmed by sequencing vectors isolated from colonies sensitive to Zeocin.

Generation of knockout and tagging constructs was performed using sequential recombineering and gateway steps as previously described [47]. Lambda red-ET recombineering was first used to introduce a bacterial selection marker amplified into the gDNA insert, such that the target gene is either deleted or prepared for 3′-end tagging. The bacterial marker was then replaced with a selection cassette for *P. berghei* in a Gateway LR Clonase reaction *in vitro*. The modified library inserts were then released from the plasmid backbone using *Not*I and used to transfect *P. berghei*.

For the pericam construct, the *P. berghei hsp70* promoter (PBANKA_071190) was first cloned using *Sac*II/*Xho*I and hsp70-F/hsp70-R primers into a *p230p* targeting vector, which also contains a resistance cassette encoding human DHFR [48]. This was followed by the pericam coding sequence [28] downstream of the promoter, using *Xho*I only and primers pc-F and pc-R. The vector was linearised using *Hind*III/*Eco*RI. The expression vector for the GFP-aequorin chimeric gene was transfected in PKG-HA and PKG^T619Q^-HA parasites as previously described [31].

Schizonts for transfection were purified from overnight cultures and transfected with 1–5 µg of linearised DNA as previously described [49]. Electroporated merozoites were injected intraveinously into a naïve mouse. Resistant parasites were selected by pyremithamine supplied in the drinking water. All transgenic parasites were cloned by limiting dilution cloning, and correct integrations of the targeting constructs were verified by diagnostic PCRs and sequencing. Negative selection of PKG-HA and PKG^T619Q^-HA parasites expressing yFCU was performed through the administration of 5 fluorocytosine via the drinking water [50], and dilution cloning was subsequently carried out allowing for further genetic modifications.

### Ca^2+^ Assays

Fluorescence measurements of purified ookinetes expressing Pericam were performed on single cells. Purified ookinetes were immobilised on Lab-Tek II chambered coverglass slides using Cell-Tak as described previously [51]. Immobilised ookinetes were imaged in PBS (Ca^2+^ and Mg^2+^ free) supplemented with 10 mM glucose. Confocal images were acquired with a LSM510 laser scanning confocal microscope (Zeiss), a Plan-Apochromat 63×/1.4 oil Ph3 objective and a LP505 filter using 405 nm or 488 nm excitation. For time lapse image acquisition, cells were imaged with a Fluar 40×/1.30 oil objective on a Zeiss Axiovert 200 M inverted microscope equipped with an AxioCam MRm camera using the multidimensional acquisition mode of the Axiovision software. Reporter fluorescence was monitored using a 21HE FURA filter set (exposure time, 50 ms) and a 38HE filter set (exposure time, 300 ms) at a rate of 1 frame per 5 s for 35 cycles. The fluorescence of individual ookinetes was subsequently analysed using the Axiovision Physiology Module.

Aequorin reconstitution and luminometric Ca^2+^ detection from purified *P. berghei* gametocytes were performed as previously described [31]. First, purified gametocytes were washed 3 times in coelenterazine loading buffer (CLB - PBS, 20 mM HEPES, 20 mM Glucose, 4 mM sodium bicarbonate, 1 mM EGTA, 0.1% w/v bovine serum albumin, pH 7.2). Reconstitution was then achieved by shaking ∼10^8^ gametocytes, in 0.5 ml CLB, supplemented with 5 µM coelenterazine for 30 min at 19°C. Loaded gametocytes were washed twice in CLB and were then suspended in 10 ml RPMI 1640, 5% FBS, 4 mM sodium bicarbonate, pH 7.2. For luminescence measurements, 150 µl of the gametocyte suspension were injected into the same volume of ookinete medium containing DMSO or C2 in a 96-well assay plate of an Orion II microplate system luminometer. For each sample 50 luminescence readings were acquired over 35 s.

Changes in the levels of intracellular free Ca^2+^ were measured using Fluo-4 (Sigma) loaded *P. falciparum* late stage schizonts that had been cultured in albumax II (Fisher Scientific) and human A+ erythrocytes (washed whole blood from the National Blood service) as previously described [4]. Excitation was measured using a SPECTRAmax microplate fluorometer. Levels of free Ca^2+^ were compared to a baseline read prior to the addition of a test reagent. Schizonts were purified magnetically (Macs; Milteny Biotec) and pelleted by centrifugation for 2 min at 500 g. Parasites were resuspended in 10× warm Ringer Buffer (122.5 mM NaCl, 5.4 mM KCl, 0.8 mM MgCl_2_, 11 mM HEPES, 10 mM D-Glucose, 1 mM NaH_2_PO_4_) to 1–2×10^8^ parasites/ml, and 2 µl of 5 mM Fluo-4 was added to 1 ml of parasite preparation. Cells were incubated with Fluo-4 at 37°C for 45 min and washed twice in warm Ringer buffer and incubated for 20 min for de-esterification followed by a further two washes. The pellet was resuspended in Ringer buffer and plated out on the bottom half of a 96-well plate. Compound dilutions were plated out on the top of the plate and the excitation of the cells measured at 20 s intervals for a period of 3 min to achieve a baseline read. The cells were then transferred onto the test compound and read for a further 5 min.

### Immunofluorescence Staining and Confocal Microscopy

Ookinete immunofluorescence assays were performed as previously described [52]. For HA staining after fixation with 3% paraformaldehyde in PBS, ookinetes were permeabilised with 0.1% Triton X-100/PBS and blocked with 2% BSA/PBS. Primary antibodies were diluted in blocking solution (rat anti-HA, 1∶200). Anti-rat Alexa488 was used as a secondary antibody together with DAPI (all from Life Technologies), all diluted 1∶200 in blocking solution. Confocal images of ookinetes were acquired with a LSM510 laser scanning confocal microscope (Zeiss).

### RNA Sequencing

Approximately 10 µg of wild-type and *gcβ* mutant RNA were extracted in duplicate from purified ookinetes using the RNeasy kit (Qiagen). Depletion of ribosomal RNA and highly abundant transcripts and sequencing library construction were performed as previously described [53]. The library was end-sequenced on an Illumina GAII instrument. TopHat [54] was used to map the Illumina reads against the *P. berghei* ANKA reference genome. Read counts and reads per kilo base per million mapped reads (RPKM) values were calculated for each gene. Differential expression was analysed using DESeq [55]. Data plots were created using R, and Artemis [56] was used to visualise transcriptome data.

### Quantitative Protein Mass Spectrometry

For the *gcβ* mutant proteome profiling, SILAC-based quantitative proteome profiling was performed essentially as previously described [19]. Briefly, 40 µg of total protein was used for each of the five replicates (20 µg from wild-type (K4/R6) and 20 µg *gcβ* mutant (K8/R10)) pooled. Protein gels were stained with colloidal Coomassie blue, and each lane was excised and cut into 12 bands that were destained and in-gel digested overnight using trypsin. Extracted peptides were suspended using 0.5% formic acid were analysed online using an Ultimate 3000 Nano/Capillary LC System (Dionex) coupled to an LTQ Orbitrap Velos hybrid mass spectrometer (Thermo Electron) equipped with a nanospray ion source. Peptides were desalted online using a micro-Precolumn cartridge (C18 Pepmap 100, LC Packings) and then separated using a 70 min RP gradient (4%–32% acetonitrile/0.1% formic acid) on a BEH C18 analytical column (1.7 µm, 75,235 µm id×10 cm, Waters) and analysed using a Top10 CID method.

For the phosphoproteomic analyses, ∼5 mg of total proteins were extracted as previously described [19] from ∼5.10^8^ purified ookinetes for each of the five and six biological replicates of the *gcβ* mutant and C2 experiments, respectively. Solubilised proteins were processed according to the FASP procedure [57],[58] and digested with Trypsin Gold (Promega). Peptides were collected by centrifugation and addition of ammonium bicarbonate and further desalted using Sep-Pak Light C18 cartridge (Waters).

IMAC purifications were performed as described previously [59], with the following modifications: Peptides were resuspended in IMAC loading buffer (50% acetonitrile, 0.1% TFA) and incubated with pre-equilibrated Phos-Select beads (Sigma) for 1 h at room temperature. The beads were then transferred to a TopTip (Glygen) and washed once with IMAC loading buffer, 1% acetic acid, and then water. Phosphopeptides were eluted with 100 µl ammonia water pH 11 and acidified using formic acid.

Phosphopeptide samples were analysed online using an Ultimate 3000 Nano/Capillary LC System (Dionex) coupled to an LTQ Orbitrap Velos hybrid mass spectrometer (Thermo Scientific) equipped with a nanospray ion source. Data were analysed using MaxQuant version 1.0.13.13 and Mascot server 2.2 (Matrix Science) and MaxQuant version 1.3.0.5 for the *gcβ* mutant experiment and C2 experiment, respectively [60]. MaxQuant processed data were searched against a combined mouse and *P. berghei* protein identified by the GeneDB database. A protein false discovery rate (FDR) of 0.01 and a peptide FDR of 0.01 were used for identification level cutoffs. Class I phosphorylation site was defined with a localisation probability of >0.75 and a score difference of >5 [18]. For the *gcβ* mutant experiment, phosphorylation sites with a *p* value for detection of significant outlier ratio ≥0.01, a ratio count ≥6, and at least a 3-fold change were defined as being regulated in the mutant. For the C2 experiment, data were filtered, and two-sample *t* testing was performed with a permutation-based FDR calculation in Perseus (1.3.0.4) as described previously [61]. Phosphorylation sites within an FDR of 0.05 with at least a 1.5-fold change were defined as being regulated by C2 treatment. See Text S1 for details of protein extraction, data acquisition, and analysis.

### Lipid Extraction and Electrospray-Mass Spectrometry Analysis

Total lipids were extracted using a modified Bligh and Dyer method from ∼5.10^8^ purified *P. berghei* ookinetes or 50 µl of packed cell volume of purified *P. falciparum* schizont per replicate. Ookinetes were first washed with PBS, suspended in 100 µl PBS, and transferred to a glass tube. Cells were lysed with 375 µl of 1∶2 (v/v) chloroform∶methanol and vortexed for 15 min. Samples were made biphasic by the addition of 125 µl of CHCl_3_ and 125 µl of H_2_O. After centrifugation at 1,000 g at room temperature for 5 min, the lower phase was transferred to a new glass vial and dried under nitrogen.

Lipids were subsequently dissolved in a mixture of chloroform∶methanol (1∶2) and acetonitrile∶iso-propanol∶water (6∶7∶2) and analysed with a Absceix 4000 QTrap, a triple quadrupole mass spectrometer equipped with a nanoelectrospray source. Samples were delivered using either thin-wall nanoflow capillary tips or a Nanomate interface in direct infusion mode (125 nl/min). The lipid extracts were analysed in both positive and negative ion modes using a capillary voltage of 1.25 kV. Tandem mass spectra (MS/MS) scanning (daughter, precursor, and neutral loss scans) was performed using nitrogen as the collision gas with collision energies between 35 and 90 V. Each spectrum encompasses at least 50 repetitive scans.

Tandem mass spectra (MS/MS) were obtained with collision energies as follows: 35–45 V, PC/SM in positive ion mode, parent-ion scanning of m/z 184; 35–55 V, PI/IPC in negative ion mode, parent-ion scanning of m/z 241; 35–65 V, PE in negative ion mode, parent-ion scanning of m/z 196; 20–35 V, PS in negative ion mode, neutral loss scanning of m/z 87; and 40–90 V, all glycerophospholipids (including PA, PG, and CL) were detected by precursor scanning for m/z 153 in negative ion mode. MS/MS daughter ion scanning was performed with collision energies between 35 and 90 V. Assignment of phospholipid species is based upon a combination of survey, daughter, precursor, and neutral loss scans, as well as previous assignments [62]. The identity of phospholipid peaks was verified using the LIPID MAPS: Nature Lipidomics Gateway (www.lipidmaps.org).

### Pathway Enrichment Analysis

The enrichment analysis was based on a list of 3,167 *P. falciparum* genes assigned to 212 metabolic pathways and functional groups present in Malaria Parasite Metabolic Pathways database (http://priweb.cc.huji.ac.il/malaria, June 2013). Annotations were transferred to *P. berghei* orthologs identified by the GeneDB database (http://www.genedb.org). Enrichment for pathways in sets of genes was calculated using a one-tailed Fisher's exact test.

## Supporting Information

Figure S1 **Workflow for genetically modifying large inserts from a** ***P. berghei*** **genomic DNA library by λ Red-ET recombinase-mediated engineering.** This protocol was developed to generate allelic exchange vectors for PKG and PI4K that mutate the gatekeeper residue or selected phosphorylation sites. Steps 1–7 can be carried out as shown to first mutate a site and then turn the modified alleles into a complementation vector. Alternatively, steps 1–4 can be used to modify an existing *Plasmo*GEM tagging vector.
(TIF)

Figure S2 **Production and genotyping of transgenic** ***P. berghei*** **lines.** (A–F) Genetic modification vectors and strategies used in this study and genotyping data for each transgenic parasite. Oligonucleotides used for PCR genotyping are indicated and agarose gels of corresponding PCR products from genotyping reactions are shown. For *pkg* and *pi4k* point mutations, sequence chromatograms of the modified sites are also shown.
(TIF)

Figure S3 **Fitness of PKG-HA and PKG^T619Q^-HA lines at different life cycle stages.** (A) Blood parasitaemia after intraperitoneal injection of 10^7^ parasites. Error bars show standard deviations from three infections. (B) Zygote-to-ookinete conversion as assessed 18 h after inducing gametogenesis *in vitro* by scoring the developmental states of 100 parasites labelled with anti–p28-Cy3 monoclonal antibody by fluorescence microscopy. Error bars show standard deviations from six cultures. (C) Number of oocysts formed per mosquito midgut. Whiskers show 2.5 and 97.5 percentiles, the box includes 50% of all values, and the horizontal line shows median values; *n* = 22 mosquitoes from two separate infection replicates. (D) Number of sporozoites per infected salivary gland. Errors show standard deviations from 44 dissected glands from two biological replicates. (E) Blood parasitaemia after mosquito bite. Error bars show standard deviations from three infections. (F) Blood stages parasitaemia after intravenous injection of 20,000 salivary gland sporozoites. Error bars as in (E).
(TIF)

Figure S4 **Molecular phenotyping of the** ***gcβ*** **mutant.** (A) Normalised transcript abundance expressed as reads per kilo base per million (RPKM) for wild-type and *gcβ* mutant ookinetes, showing no gross differences in transcription levels. A representative experiment of two replicates is shown. (B) Coverage plot showing mRNA sequencing reads mapping to a 3′ fragment of the disrupted *gcβ* gene of the *gcβ* mutant. Translation of partial mRNAs from the disrupted gene would be predicted to result in a nonfunctional protein lacking a complete cyclase domain, which explains the detection of peptides from GCβ in the proteome of the mutant (see, e.g., Figure 2B). (C) Normalised protein ratios deduced from five biological replicates are plotted against the heavy/medium ratio counts for each protein.
(TIF)

Figure S5 **Effect of zaprinast and C2 on phospholipids of** ***P. berghei*** **ookinetes,** ***P. falciparum*** **schizont–infected erythrocytes, and uninfected erythrocytes.** (A) Total phospholipid analysis of *P. berghei* ookinetes. (B) Phosphorylated PI analysis in the presence of C2 of *P. berghei* ookinetes expressing PKG-HA or PKG^T619Q^-HA over a m/z range of 1,000–1,300. (C) Phospholipid analysis of *P. falciparum* schizont–infected erythrocytes over an m/z range of 600–1,000. (D) Phospholipid analysis of human uninfected RBCs in negative ion mode (600–1,000 m/z, left panel; 1,000–1,300 m/z, right panel), showing low or undetectable levels of PIPs.
(TIF)

Figure S6 **Characterisation of Ca^2+^ reporters in** ***P. berghei*** **ookinetes and** ***P. falciparum*** **schizonts.** (A) Confocal immunofluorescence images of a live ookinete expressing the dual excitation ratiometric calcium reporter pericam showing unbound reporter from excitation at 405 nm and bound reporter from excitation at 488 nm. Scale bar, 5 µm. (B) Fluorescence response of wild-type purified ookinetes expressing pericam after the addition of 0.5 µM ionomycin (t = 20 s), followed by 10 mM calcium (t = 60 s). Fluorescence was normalised as follow: ΔF = (F_n_−F_20_)/F_20_, in which F_n_ is the fluorescence at t = *n* s and F_20_ is the reference time t = 20 s before addition of C2. Error bars indicate the standard error of the mean from three independent replicates each representative of 10 ookinetes. (C) Fluorescence response of synchronised *P. falciparum* 3D7 schizonts loaded with Fluo-4 to varying concentrations of zaprinast and to the A23187 ionophore.
(TIF)

Table S1 **Transcriptome, proteome, and phosphoproteome analyses of altered cGMP signalling in ookinetes.** The table displays the full list of transcripts, proteins, and phosphorylation sites that were detected in the *gcβ* or C2 experiments. Fold changes are indicated as FC, and S(A) is the *p* value for detection of significant outlier ratio.
(XLS)

Table S2 **Phosphorylation sites significantly regulated in the** ***gcβ*** **mutant and C2 experiments.** The table displays the list of phosphorylation ratios for significantly regulated phosphorylated sites in the *gcβ* and the C2 experiments, respectively. Annotation from the Malaria Parasite Metabolic Pathway database is indicated.
(XLSX)

Table S3 **Oligonucleotides used in this study.** The table displays the list of oligonucleotides used in this study.
(XLS)

Text S1 **Supplementary methods.**
(DOCX)

## Abbreviations

ARF: ADP-ribosylation factor
ARF-GAP: ADP-ribosylation factor GTPase activating protein
ARF-GEF: ADP-ribosylation factor guanine exchange factor
CDPK: Ca^2+^-dependent protein kinase
cGMP: 3′-5′-cyclic guanosine monophosphate
GC: guanylyl cyclase
IMAC: immobilised metal ion chromatography
IP_3_: inositol (1,4,5)-trisphosphate
PDE: phosphodiesterase
PI: phosphatidyl-1D-*myo*-inositol
PI(4,5)P_2_: phosphatidylinositol (4,5)-bisphosphate
PI4K: phosphatidylinositol 4-kinase
PI4P: phosphatidylinositol 4-phosphate
PI-PLC: PI-specific phospholipase C
PIP5K: phosphatidylinositol 5-kinase
PKG: cGMP-dependent protein kinase G
SILAC: stable isotope labelling in culture
