# Supplementary figures and images for: Phosphoinositide Metabolism Links cGMP-Dependent Protein Kinase G to Essential Ca2+ Signals at Key Decision Points in the Life Cycle of Malaria Parasites
Source: PLoS Biol. 2014 Mar 4;12(3):e1001806. doi: 10.1371/journal.pbio.1001806 (PMC3942320; doi:10.1371/journal.pbio.1001806)

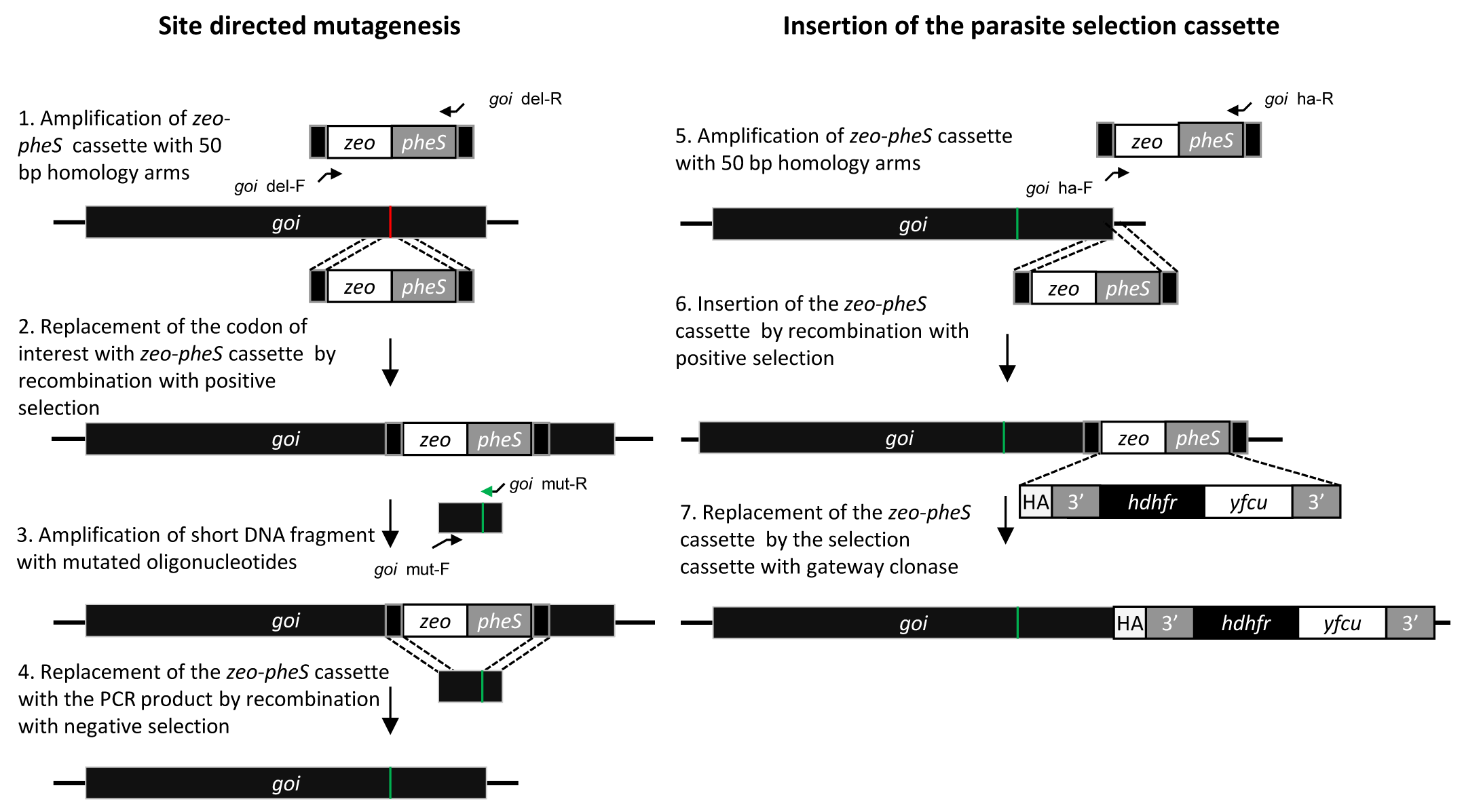

Supplement: Figure S1 — Workflow for genetically modifying large inserts from a P. berghei genomic DNA library by λ Red-ET recombinase-mediated engineering. This protocol was developed to generate allelic exchange vectors for PKG and PI4K that mutate the gatekeeper residue or selected phosphorylation sites. Steps 1–7 can be carried out as shown to first mutate a site and then turn the modified alleles into a complementation vector. Alternatively, steps 1–4 can be used to modify an existing PlasmoGEM tagging vector. (TIF) [file pbio.1001806.s001.tif]

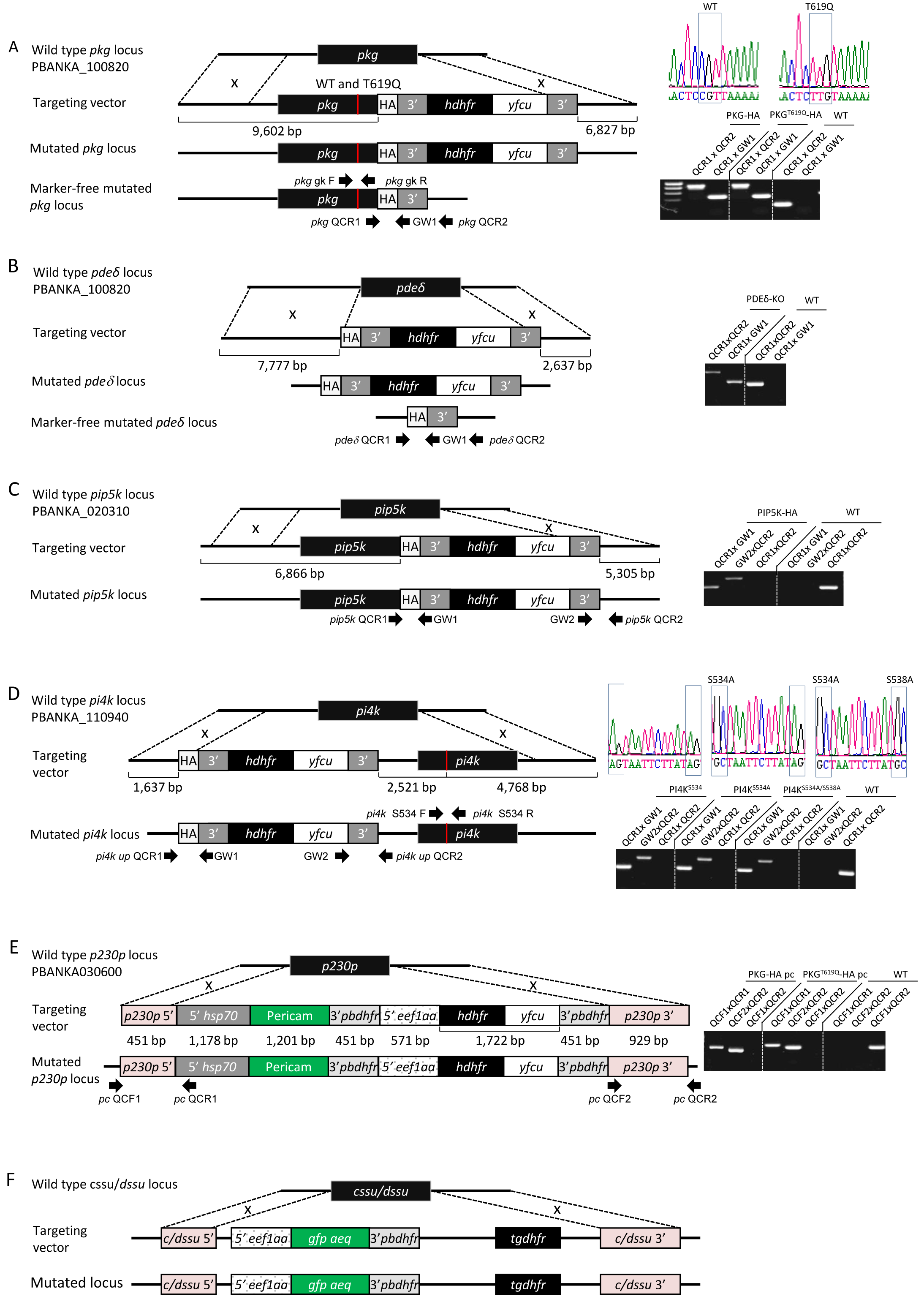

Supplement: Figure S2 — Production and genotyping of transgenic P. berghei lines. (A–F) Genetic modification vectors and strategies used in this study and genotyping data for each transgenic parasite. Oligonucleotides used for PCR genotyping are indicated and agarose gels of corresponding PCR products from genotyping reactions are shown. For pkg and pi4k point mutations, sequence chromatograms of the modified sites are also shown. (TIF) [file pbio.1001806.s002.tif]

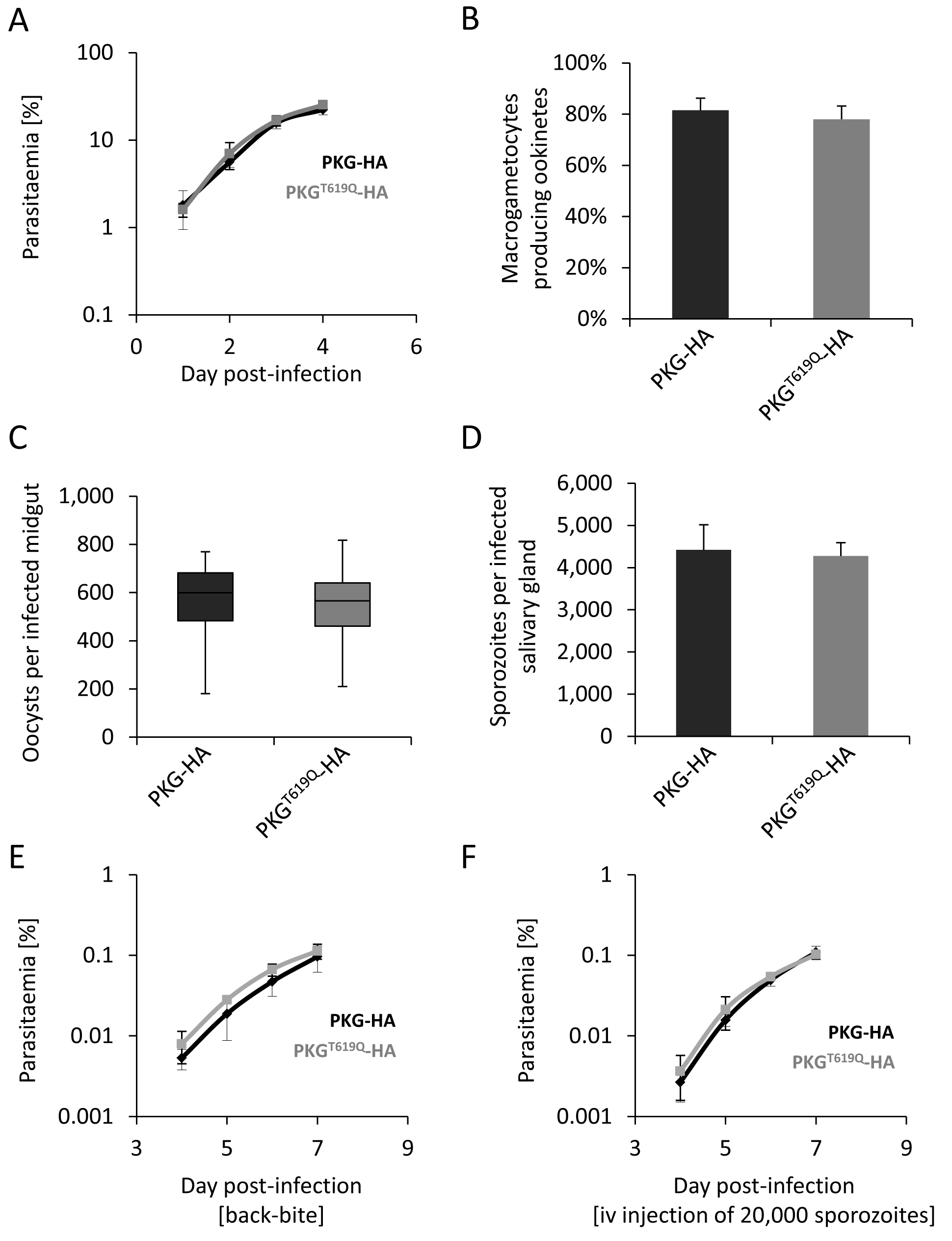

Supplement: Figure S3 — Fitness of PKG-HA and PKGT619Q-HA lines at different life cycle stages. (A) Blood parasitaemia after intraperitoneal injection of 107 parasites. Error bars show standard deviations from three infections. (B) Zygote-to-ookinete conversion as assessed 18 h after inducing gametogenesis in vitro by scoring the developmental states of 100 parasites labelled with anti–p28-Cy3 monoclonal antibody by fluorescence microscopy. Error bars show standard deviations from six cultures. (C) Number of oocysts formed per mosquito midgut. Whiskers show 2.5 and 97.5 percentiles, the box includes 50% of all values, and the horizontal line shows median values; n = 22 mosquitoes from two separate infection replicates. (D) Number of sporozoites per infected salivary gland. Errors show standard deviations from 44 dissected glands from two biological replicates. (E) Blood parasitaemia after mosquito bite. Error bars show standard deviations from three infections. (F) Blood stages parasitaemia after intravenous injection of 20,000 salivary gland sporozoites. Error bars as in (E). (TIF) [file pbio.1001806.s003.tif]

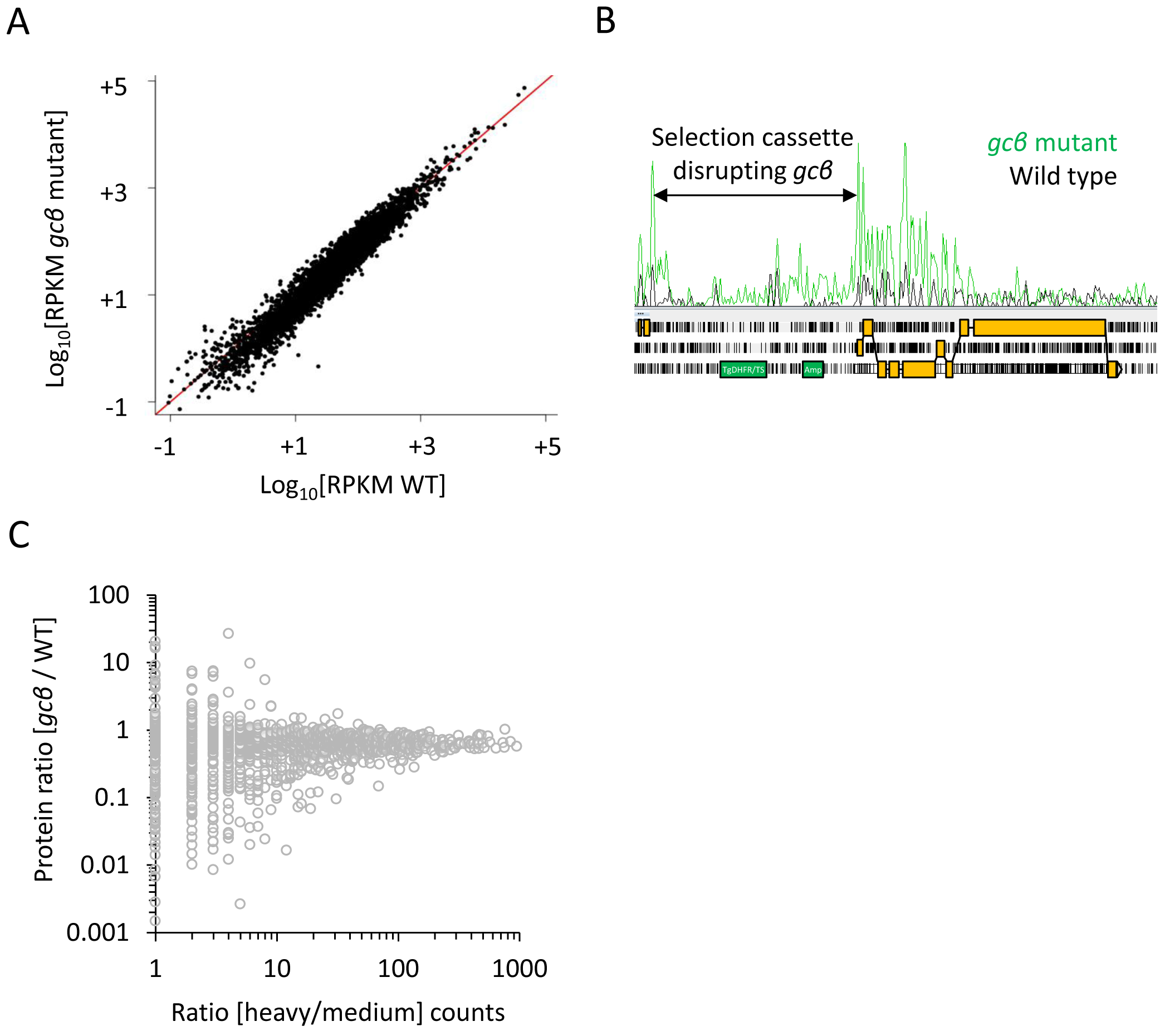

Supplement: Figure S4 — Molecular phenotyping of the gcβ mutant. (A) Normalised transcript abundance expressed as reads per kilo base per million (RPKM) for wild-type and gcβ mutant ookinetes, showing no gross differences in transcription levels. A representative experiment of two replicates is shown. (B) Coverage plot showing mRNA sequencing reads mapping to a 3′ fragment of the disrupted gcβ gene of the gcβ mutant. Translation of partial mRNAs from the disrupted gene would be predicted to result in a nonfunctional protein lacking a complete cyclase domain, which explains the detection of peptides from GCβ in the proteome of the mutant (see, e.g., Figure 2B). (C) Normalised protein ratios deduced from five biological replicates are plotted against the heavy/medium ratio counts for each protein. (TIF) [file pbio.1001806.s004.tif]

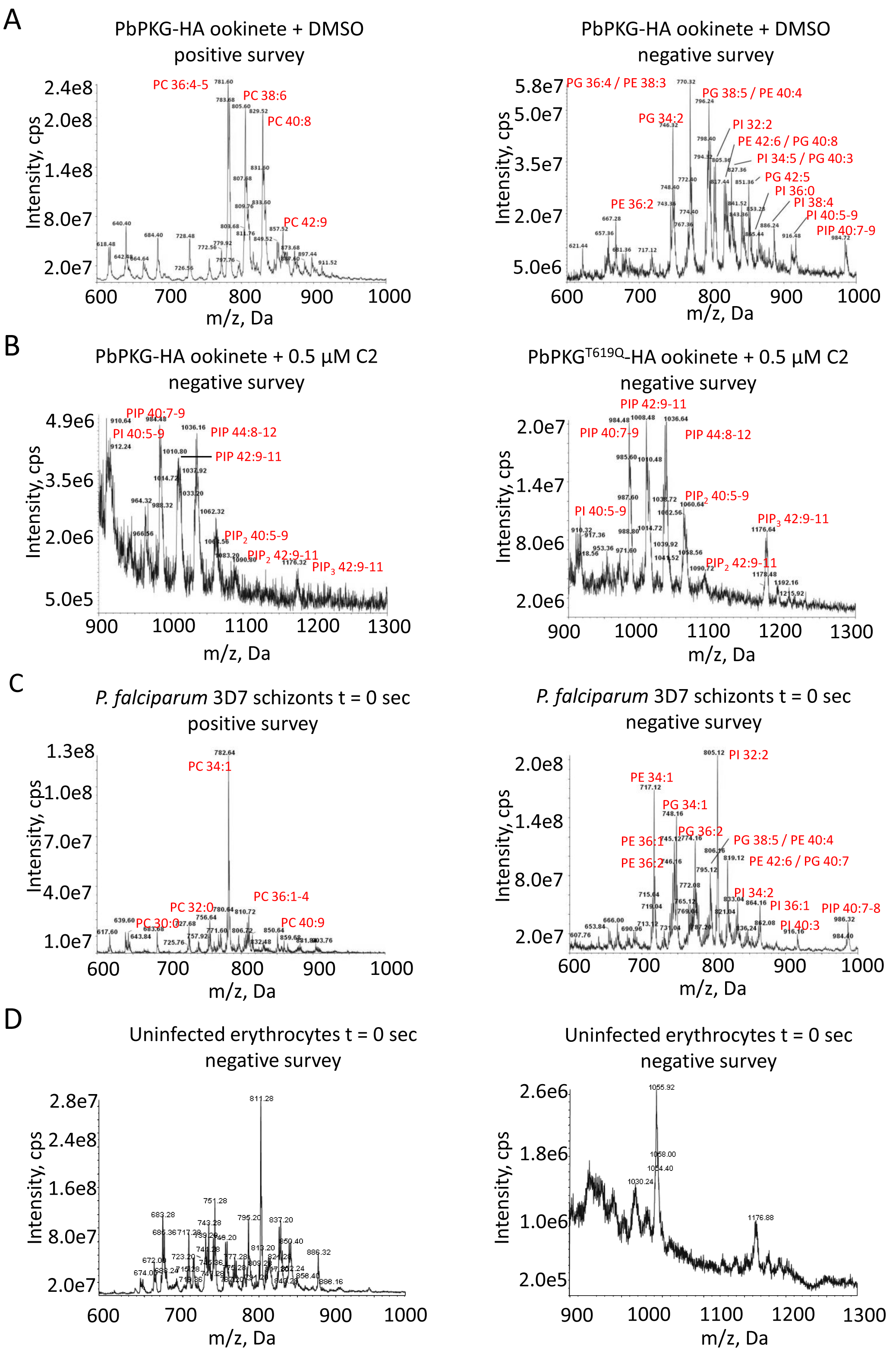

Supplement: Figure S5 — Effect of zaprinast and C2 on phospholipids of P. berghei ookinetes, P. falciparum schizont–infected erythrocytes, and uninfected erythrocytes. (A) Total phospholipid analysis of P. berghei ookinetes. (B) Phosphorylated PI analysis in the presence of C2 of P. berghei ookinetes expressing PKG-HA or PKGT619Q-HA over a m/z range of 1,000–1,300. (C) Phospholipid analysis of P. falciparum schizont–infected erythrocytes over an m/z range of 600–1,000. (D) Phospholipid analysis of human uninfected RBCs in negative ion mode (600–1,000 m/z, left panel; 1,000–1,300 m/z, right panel), showing low or undetectable levels of PIPs. (TIF) [file pbio.1001806.s005.tif]

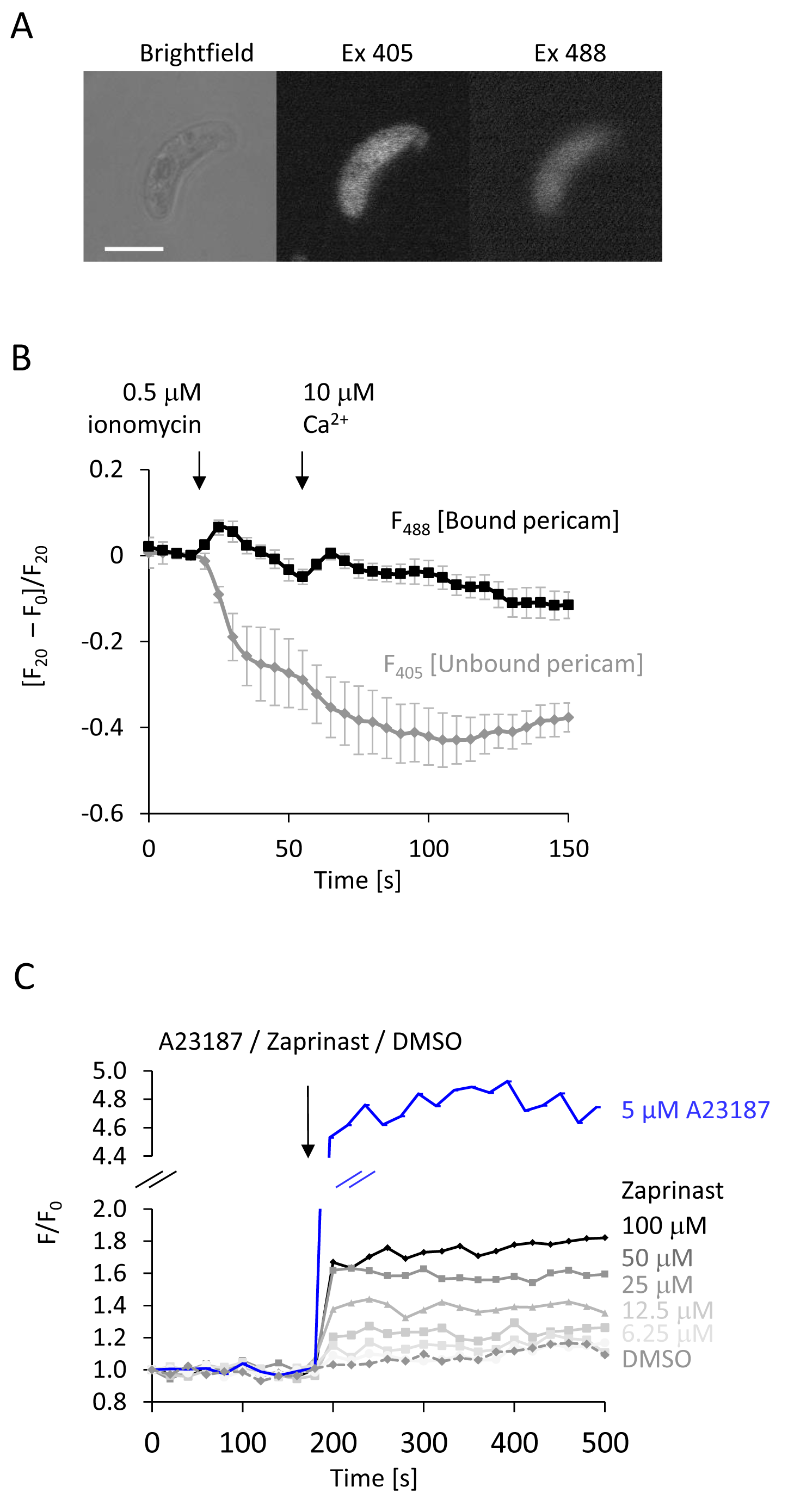

Supplement: Figure S6 — Characterisation of Ca2+ reporters in P. berghei ookinetes and P. falciparum schizonts. (A) Confocal immunofluorescence images of a live ookinete expressing the dual excitation ratiometric calcium reporter pericam showing unbound reporter from excitation at 405 nm and bound reporter from excitation at 488 nm. Scale bar, 5 µm. (B) Fluorescence response of wild-type purified ookinetes expressing pericam after the addition of 0.5 µM ionomycin (t = 20 s), followed by 10 mM calcium (t = 60 s). Fluorescence was normalised as follow: ΔF = (Fn−F20)/F20, in which Fn is the fluorescence at t = n s and F20 is the reference time t = 20 s before addition of C2. Error bars indicate the standard error of the mean from three independent replicates each representative of 10 ookinetes. (C) Fluorescence response of synchronised P. falciparum 3D7 schizonts loaded with Fluo-4 to varying concentrations of zaprinast and to the A23187 ionophore. (TIF) [file pbio.1001806.s006.tif]
