# Supplementary material for: Phosphoinositide Metabolism Links cGMP-Dependent Protein Kinase G to Essential Ca2+ Signals at Key Decision Points in the Life Cycle of Malaria Parasites
Source: PLoS Biol. 2014 Mar 4;12(3):e1001806. doi: 10.1371/journal.pbio.1001806 (PMC3942320; doi:10.1371/journal.pbio.1001806)
Supplement: Text S1 — Supplementary methods. (DOCX) [file pbio.1001806.s010.docx]

**Supplementary methods**

**Protein extraction, digestion and clean up**

Parasite pellets were re-suspended in extraction buffer (4% SDS, 0.1 M DTT, 0.1 M Tris pH 8, 0.5 mM PMSF, 2 μg/ml Aprotinin/Leupeptin, 20 μM ZnCl_2_ and 25 mM Sodium fluoride), homogenised in a 2 ml dounce homogeniser with 25 strokes. The sample was heated for 5 min at 97 °C, DNA was sheared by passing the lysate through a fine gauge needle and insoluble material was pelleted by centrifugation at 14,000 rpm for 10 min. The insoluble pellet was further extracted by addition of urea (once cooled to room temperature) to a final concentration of 8 M. Homogenisation and centrifugation steps were repeated and supernatants containing solubilized protein from both extractions were pooled and applied to a pre-washed Amicom-15 centrifugal filter unit (30 kDa MWCO) (Millipore). The protein sample was processed according to the FASP procedure [[1](#_ENREF_1),[2](#_ENREF_2)] in which SDS was removed by buffer exchange with urea and cysteine alkylation was performed in situ with iodoacetamide and proteins were digested with Trypsin Gold (Promega) for 4 h at 37°C at an enzyme substrate ratio of 1:20 with a urea concentration of 1 M and 100 mM ammonium bicarbonate. Peptides were collected by centrifugation and addition of 100 mM ammonium bicarbonate to the upper chamber of the unit and further centrifugation. Collected peptides were adjusted to a 0.4 % TFA and desalted using Sep-Pak® Light C18 cartridge (Waters) and dried down using a SpeedVac (Thermo Scientific).

**LC-MS/MS analysis**

*gcβ* mutant phosphoproteome

Phosphopeptides from IMAC purifications (5 biological replicates) were split and each analysed by 3 replicate LC-MS/MS analyses. Phosphopeptide samples were analysed online using an Ultimate 3000 Nano/Capillary LC System (Dionex) coupled to an LTQ Orbitrap Velos hybrid mass spectrometer (Thermo Scientific) equipped with a nanospray ion source. Peptides were desalted on-line using a micro-Precolumn cartridge (C18 Pepmap 100, LC Packings) (with 0.5% acetic acid) and then separated using a 200 min RP gradient (4-30 % acetonitrile/0.1 % formic acid) on an Acclaim PepMap100 C18 analytical column (3 μm, 75 μm id x 50 cm) (Dionex) with a flow rate of 0.3 μl/min. The mass spectrometer was operated in standard data dependent acquisition mode controlled by Xcalibur 2.1. The instrument was operated with a cycle of one MS (in the Orbitrap) acquired at a resolution of 60,000 at m/z 400, with the top 10 most abundant multiply-charged (2+ and higher) ions in a given chromatographic window were subjected to CID fragmentation in the linear ion trap. An FTMS target value of 10^6^ and an ion trap MSn target value of 5,000 were used. Dynamic exclusion was enabled with a repeat duration of 45 sec with an exclusion list of 500 and exclusion duration of 45 sec. Lock mass of 445.120025 was enabled for all experiments.

C2 phosphoproteome

Phosphopeptides from IMAC purifications (6 biological replicates) were split and each analysed by 2 replicate LC-MS/MS analyses. Phosphopeptide samples were analysed online using an Ultimate 3000 RSLC Nano LC System (Dionex) coupled to an LTQ Orbitrap Velos hybrid mass spectrometer (Thermo Scientific) equipped with an Easy-Spray (Thermo Scientific) ion source. Peptides were desalted on-line using a capillary trap column (Acclaim Pepmap100, 100 μm, 75 μm x 2 cm, C18, 5 μm - Thermo Scientific) with 0.5 % acetic acid and then separated using a 240 min RP gradient (4-30 % acetonitrile/0.1 % formic acid) on an Acclaim PepMap100 RSLC C18 analytical column (2 μm, 75 μm id x 50 cm - Thermo Scientific) with a flow rate of 0.3 μl/min. The mass spectrometer was operated in standard data dependent acquisition mode controlled by Xcalibur 2.2. The instrument was operated with a cycle of one MS (in the Orbitrap) acquired at a resolution of 60,000 at m/z 400, with the top 10 most abundant multiply-charged (2+ and higher) ions in a given chromatographic window were subjected to CID fragmentation in the linear ion trap. An FTMS target value of 10^6^ and an ion trap MSn target value of 5,000 were used. Dynamic exclusion was enabled with a repeat duration of 30 sec with an exclusion list of 500 and exclusion duration of 60 sec. Lock mass of 445.120025 was enabled for all experiments.

**Protein mass spectrometry data analysis**

SILAC labelled data was analysed using MaxQuant version 1.0.13.13 and Mascot server 2.2 (Matrix Science) and MaxQuant version 1.3.0.5 for the *gcβ* mutant experiment and C2 experiment respectively [[3](#_ENREF_3)]. MaxQuant processed data was searched against a combined mouse and *P. berghei* protein sequence database using the following search parameters: trypsin with a maximum of 2 missed cleavages, 7 ppm for MS mass tolerance, 0.5 Da for MS/MS mass tolerance, with acetyl (Protein N-term) and oxidation (M) (as well as phospho STY set as variable modifications) and carbamidomethyl (C) as a fixed modification. A protein false discovery rate (FDR) of 0.01 and a peptide FDR of 0.01 were used for identification level cut offs. Class I phosphorylation site were defined with a localisation probability of >0.75 and a score difference of >5 [[4](#_ENREF_4)].

For the *gcβ* mutant experiment, phosphorylation sites with a p-value for detection of significant outlier ratio ≥ 0.01, a ratio count ≥ 6, and at least a 2 fold change were defined as being regulated in the mutant. Match between runs with a 2 minute retention time window was enabled for the analysis of C2 experiment data. Peptide intensity values were calculated by MaxQuant for up to a total of 24 samples (6 biological replicates each for compound 2 treated and untreated and 2 technical replicates of each) and outliers were removed prior to averaging intensities from technical replicates. Data was filtered so that at least 8 valid intensity values were present (8 out of 12 biological replicates), log2 transformed and two sample t-testing was performed with a permutation based FDR calculation in Perseus (1.3.0.4). Phosphorylation sites within an FDR of 0.05 with at least a 1.5-fold change were defined as being regulated by C2 treatment.

**Supplementary references**

1. Manza LL, Stamer SL, Ham AJ, Codreanu SG, Liebler DC (2005) Sample preparation and digestion for proteomic analyses using spin filters. Proteomics 5: 1742-1745.

2. Wisniewski JR, Zougman A, Nagaraj N, Mann M (2009) Universal sample preparation method for proteome analysis. Nat Methods 6: 359-362.

3. Cox J, Mann M (2008) MaxQuant enables high peptide identification rates, individualized p.p.b.-range mass accuracies and proteome-wide protein quantification. Nat Biotechnol 26: 1367-1372.

4. Olsen JV, Blagoev B, Gnad F, Macek B, Kumar C, et al. (2006) Global, *in vivo*, and site-specific phosphorylation dynamics in signaling networks. Cell 127: 635-648.
